# Role of CBP and SATB-1 in Aging, Dietary Restriction, and Insulin-Like Signaling

**DOI:** 10.1371/journal.pbio.1000245

**Published:** 2009-11-17

**Authors:** Minhua Zhang, Michal Poplawski, Kelvin Yen, Hui Cheng, Erik Bloss, Xiao Zhu, Harshil Patel, Charles V. Mobbs

**Affiliations:** 1 Department of Neuroscience, Mount Sinai School of Medicine, New York, New York, United States of America; 2 Department of Geriatrics, Mount Sinai School of Medicine, New York, New York, United States of America; The Salk Institute, United States of America

## Abstract

How dietary restriction (DR) increases lifespan and decreases disease burden are questions of major interest in biomedical research. Here we report that hypothalamic expression of CREB-binding protein (CBP) and CBP-binding partner Special AT-rich sequence binding protein 1 (SATB-1) is highly correlated with lifespan across five strains of mice, and expression of these genes decreases with age and diabetes in mice. Furthermore, in *Caenorhabditis elegans*, *cbp-1* is induced by bacterial dilution DR (bDR) and the *daf-2* mutation, and *cbp-1* RNAi specifically in adults completely blocks lifespan extension by three distinct protocols of DR, partially blocks lifespan extension by the *daf-2* mutation but not of cold, and blocks delay of other age-related pathologies by bDR. Inhibiting the *C. elegans* ortholog of SATB-1 and CBP-binding partners *daf-16* and *hsf-1* also attenuates lifespan extension by bDR, but not other protocols of DR. In a transgenic Aβ42 model of Alzheimer's disease, *cbp-1* RNAi prevents protective effects of bDR and accelerates Aβ42-related pathology. Furthermore, consistent with the function of CBP as a histone acetyltransferase, drugs that enhance histone acetylation increase lifespan and reduce Aβ42-related pathology, protective effects completely blocked by *cbp-1* RNAi. Other factors implicated in lifespan extension are also CBP-binding partners, suggesting that CBP constitutes a common factor in the modulation of lifespan and disease burden by DR and the insulin/IGF1 signaling pathway.

## Abstract 2

Increased transcriptional complex activity, or pharmacological mimics of increased complex activity, predict lifespan in mice and mediate the protective effects of dietary restriction during aging.

## Author Summary

The simple manipulation of dietary restriction (DR) (reduction of caloric intake by about 30% in rodents) produces robust increases in lifespan and slows the development of almost all age-related diseases, including cancer and neurological diseases. This relationship between dietary restriction and longevity is observed in most models in which the effect of DR has been tested. Thus, understanding how DR produces its protective mechanisms would have potentially profound implications for the treatment of age-related diseases, including possibly the development of a “magic bullet” for these diseases. In the present study we have discovered that DR induces a transcription factor, CBP, and additional factors that work with CBP to control the expression of other genes involved in determination of lifespan. When we blocked the DR-mediated increase in CBP and associated factors, we blocked all the protective effects of DR on lifespan extension, on the slowed rate of aging, and on protection against pathology in a model of Alzheimer's disease. Further, in mice expression of CBP and a CBP-interacting factor positively predicted lifespan, and expression of both factors decreased with age and in diabetes. Finally, pharmacological manipulations that mimicked enhanced CBP activity increased lifespan and reduced pathology in a model of Alzheimer's disease.

## Introduction

Elucidation of mechanisms mediating lifespan extension and reduction of disease burden, including cancer and neurodegenerative diseases, by DR is a major goal of aging research [1]. Recent studies have implicated sirtuins [2], SKN-1 [3], SMK-1 and PHA-4/Foxa [4], AMPK [5], RHEB-1 [6], daf-16/Fox1a [5], and HSF-1 [7] in mediating lifespan extension by some, but not all [8],[9], protocols of DR in *Caenorhabditis elegans*. However, a role for expression of these genes in mammalian lifespan has not been addressed, nor, with rare exceptions [7], has a role for expression of these genes in reduction of age-related pathologies by DR. The purpose of the present studies was to discover genes whose expression predicts lifespan and whose expression decreases with age and disease in mammals, whose expression is induced by DR, and whose inhibition attenuates life extension by several distinct protocols of DR. We report that among genes implicated in lifespan extension by DR or the insulin-like signaling pathway, only CBP meets these criteria.

## Results

### Expression of CBP and SATB-1 Predicts Lifespan and Decreases With Age and Diabetes in Mice

Since hypothalamic neurons mediate physiological responses to nutritional deprivation, we hypothesized that hypothalamic gene expression may play a role in mediating lifespan extension by DR [10], a hypothesis supported by the observation that two neurons mediate protective effects of DR in *C. elegans* [3]. In a small microarray survey to discover candidates that may mediate protective effects of DR, the only transcription factor we corroborated to be induced by nutritional deprivation in mouse hypothalamus was the transcription factor CBP/p300-interacting transactivator with Glu/Asp-rich carboxy-terminal domain 1 (CITED-1) [11]. In a larger microarray survey, we observed over 40 transcription factors induced by nutritional deprivation in mouse hypothalamus, among the most prominent of which were CBP and its co-factors.

**Figure 1:**
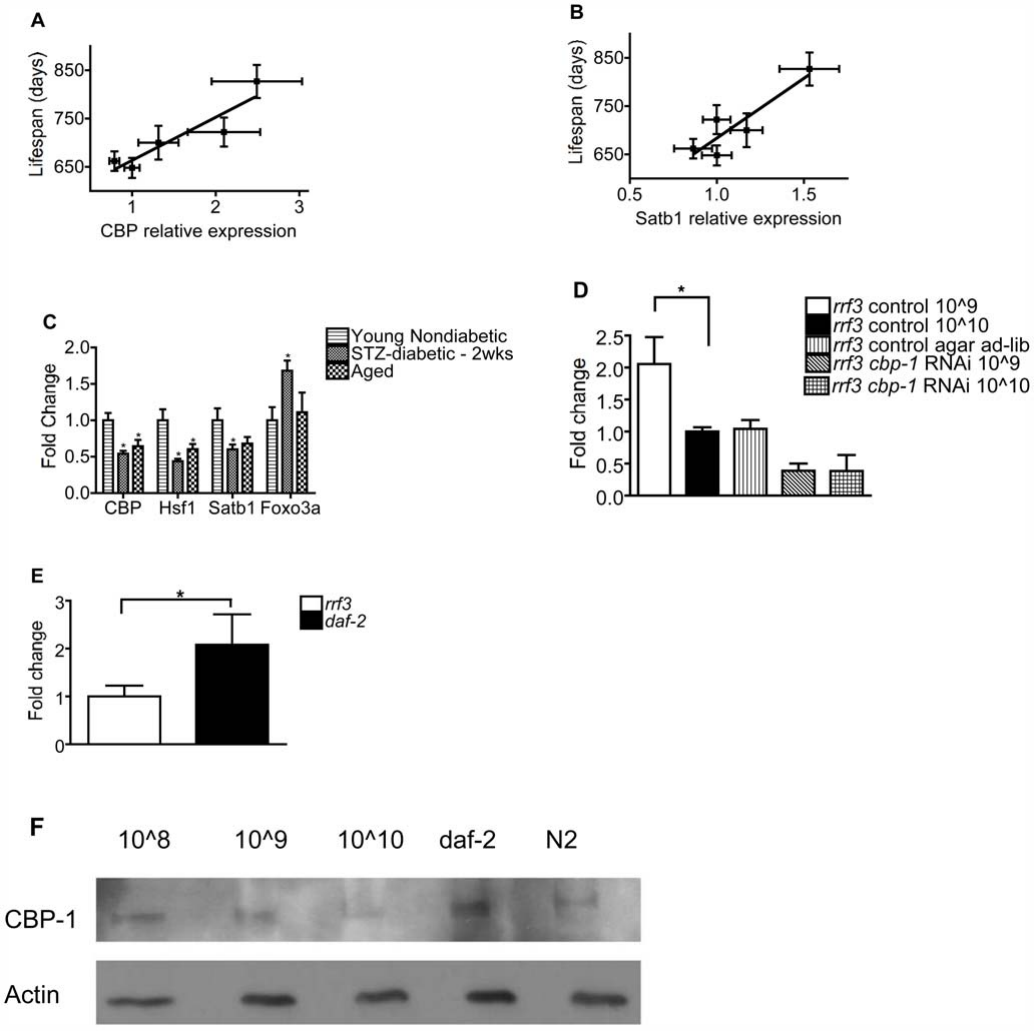
Expression of CBP and SATB-1 predicts lifespan, is reduced with aging and diabetes, and is induced by DR and the *Daf-2* mutation. Hypothalamic mRNA, assessed by q-PCR, of (A) *cbp* (*n* = 10/group, *p* = 0.027, *R* ^2^ = 84%) and (B) *satb-1* (*n* = 10/group, *p* = 0.036, *R* ^2^ = 81%) correlates with average lifespan across five mouse strains (BALB/cByJ, A/J, C3H/HeJ, DBA/2J, and C57Bl/6J, in order of increasing lifespan). (C) Cortical mRNA of CBP, SATB-1, HSF-1, and FOXO3A in young (10–12 wk), aged (18–19 mo), and diabetic male C57Bl/6J mice. Data are presented as mean ± SEM (*n* = 7–14/group, *p<0.05). *cbp-1* mRNA is (D) induced by bDR (10̂9 bacteria/ml) and inhibited by *cbp-1* RNAi, and (E) induced by the *daf-2* hypomorphic allele. (F) CBP-1 protein, assessed by immunoblotting, is induced by DR (10̂9 bacteria/ml) and the *daf-2* mutation.

Hypothesizing that expression of genes mediating lifespan extension by DR may also predict lifespan under ad lib fed conditions, we screened over 40 genes, including CBP and genes otherwise implicated in lifespan extension, to detect genes whose hypothalamic expression predicts lifespan across five strains of mice (BALB/cByJ, A/J, C3H/HeJ, DBA/2J, and C57Bl/6J) in order of increasing lifespan, based on published lifespan data (see Methods) [12],[13]. Hypothalamic expression of CBP and SATB1 were highly and positively correlated with lifespan (Figure 1A and 1B), accounting for 84% and 81%, respectively, of lifespan variance. These results were corroborated in a second set of mice purchased over 2 y after the first strain comparison study, with essentially identical results. In contrast, hypothalamic expression of other genes implicated in aging, including mammalian orthologs to sirtuins, *daf-16/FoxO3a*, *pha-4/FoxA*, *rheb-1*, *hsf-1*, *skn-1/NFE2*, *hif-1*, and *aak-2/ampk*, did not correlate significantly with lifespan. Similarly, cortical expression of CBP, SATB-1, and HSF-1 but not other genes implicated in influencing lifespan (e.g., the *daf-16* ortholog FOXO3A) decreased with age and diabetes, a disease that mimics many effects of aging (Figure 1C) [14].

To address the functional role of these genes in determining senescent phenotypes using RNA interference (RNAi), we extended these studies to *C. elegans*. Expression of *cbp-1*, the *C. elegans* ortholog of mammalian CBP [15], was induced in *C. elegans* by both DR (produced by dilution of bacteria to optimize lifespan [8] bDR) and mutation of the insulin-like receptor (*daf-2*) (Figure 1D, 1E, 1F). Furthermore, *cbp-1* RNAi completely blocked induction of the gene by bacterial dilution and reduced *cbp-1* expression to about half the level of the ad lib bacterial concentration (Figure 1D); the inhibition was similar at both ad lib and diluted bacterial concentrations (Figure 1D). In contrast to mouse hypothalamus, we did not observe decreased expression of *cbp-1* in whole *C. elegans* during aging.

### *cbp*-1 RNAi Completely Blocks Lifespan Extension by Three Protocols of DR in *C. elegans*

Three classes of protocols for DR have been used to extend lifespan in *C. elegans*: “axenic” or “dietary deprivation” dietary restriction with no bacteria [16],[17],[5.], genetic reduction of feeding rate (e.g., by mutation in the *eat-2* gene [18]), or optimized bacterial dilution in liquid media (bDR) [19],[3],[8]. Lifespan extension by different DR protocols requires different sets of genes [9], although it has been argued that the optimized bacterial dilution method provides the most valid protocol [3],[8]. We therefore used one protocol from each class of DR to assess the role of *cbp-1* in lifespan extension by DR. First, entailing axenic liquid media, worms were maintained on standard solid agar plates in which bacteria (RNAi-bearing or control) were present on adult days 1–5, then transferred to liquid axenic media with no bacteria for the remainder of the study [16]. This protocol produces lifetime inhibition of the target gene (unpublished data), but to our knowledge no genes have previously been implicated in lifespan extension by axenic media [9]. Second, worms expressing the *eat-2* mutation were maintained on standard solid media with bacteria; extension of lifespan by this protocol requires the activity of several genes implicated in aging including *hsf-1* but not *daf-16*/*foxO3a* or *aak-2*/*ampk* [9]. Finally, worms were maintained in liquid media in which bacteria were diluted for optimum lifespan (bDR) [19],[3],[8]. The bacterial dilution producing the greatest average lifespan was determined by serial bacterial dilution, and the optimum concentration for longest lifespan was 10̂9 bacteria/ml (Figure S1), similar to the optimum concentration previously reported [19]. Two other bacteria concentrations, 10̂8 and 10̂10 cells/ml, both of which reduced lifespan compared to 10̂9 cells/ml (Figure S1), were used for comparison. In all studies worms were fed bacteria expressing control (L4440) or dsRNAi constructs in the adult phase only. The RNAi-sensitive rrf-3 strain [20] used in some of these studies exhibited the same average lifespan and lifespan extension as exhibited in the control N2 strain (Table S1).

**Figure 2:**
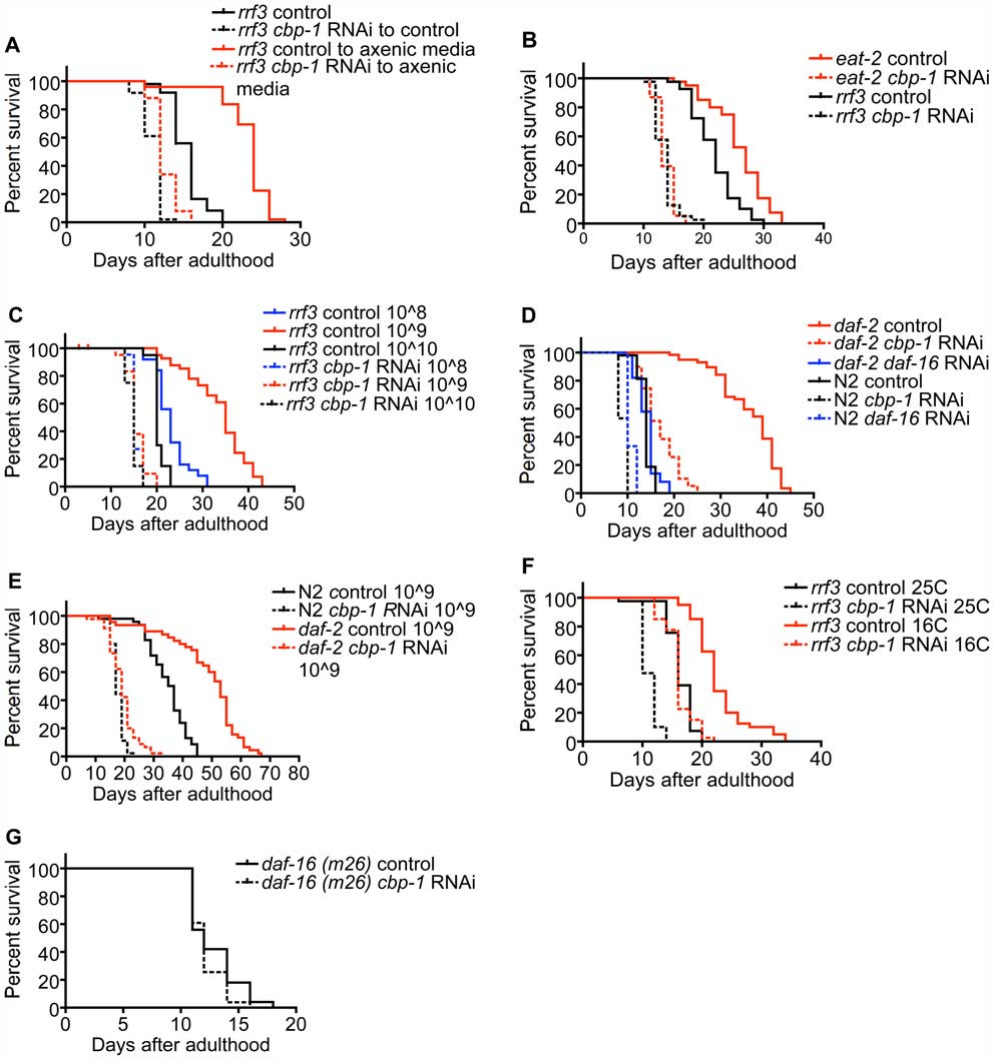
*Cbp-1* RNAi blocks life extension by DR produced by three protocols. (A) *A*xenic media; adult *rrf3* worms were fed bacteria expressing control L4440 or *cbp-1* dsRNA for 5 d, then transferred to liquid axenic media. (B) *eat-2* mutation; adult *eat-2* mutant worms (ad1113) were fed bacteria expressing control L4440 or *cbp-1* dsRNA. (C) Bacterial dilution (bDR); adult worms were grown in liquid media at three concentrations of bacteria expressing control L4440 or *cbp-1* dsRNA. (D) *daf-2* mutant or wild-type worms under standard conditions were fed bacteria expressing control L4440 or *cbp-1* dsRNA (*cbp-1* RNAi only partly blocks lifespan extension by the *daf-2* mutation; *p*<0.05). (E) *daf-2* mutant worms under optimum bacterial concentration (bDR) were fed bacteria expressing control L4440 or *cbp-1* dsRNA (*cbp-1* RNAi blocks lifespan extension by the both bDR and the *daf-2* mutation in these conditions). (F) Cold-induced longevity; adult worms were maintained under standard conditions at either 25 C or 16 C and fed bacteria expressing control L4440 or *cbp-1* dsRNA (40% extension in controls versus 44% extension in the *cbp-1* RNAi group; effect not significant). (G) *cbp-1* or control RNAi in *daf-16* hypomorphic worms (effect not significant).

The axenic media protocol increased average lifespan in worms fed control bacteria by about 50%, an extension of lifespan completely blocked by *cbp-1* RNAi (Figure 2A, Table S1). The *eat-2* mutation increased lifespan by about 20%, an extension also completely blocked by *cbp-1* RNAi (Figure 2B, Table S1). At the optimum bacterial concentration (10̂9 bacteria/ml), lifespan increased by about 65% relative to ad lib concentration (10̂10) bacteria/ml, an extension also completely blocked by *cbp-1* RNAi (Figure 2C, Table S1). Thus, inhibiting *cbp-1* by only 50% specifically in the adult phase (Figure 1D) completely blocked lifespan extension by three distinct protocols of DR.

To assess the specificity of the reversal of DR-induced lifespan extension by *cbp-1* RNAi, we assessed if *cbp-1* RNAi, like *daf-16* RNAi, would block lifespan extension by the *daf-2* mutation. Under standard conditions the *daf-2* mutation increased average lifespan by 157% (Figure 2D, Table S1), consistent with previous reports [21]. While in contrast to DR *cbp-1* RNAi did not completely block lifespan extension by the *daf-2* mutation, it did substantially reduce the degree of lifespan extension by the *daf-2* mutation (Figure 2D, Table S1). While *daf-16* RNAi and *cbp-1* RNAi reduced average lifespan in ad lib conditions to about the same extent, *cbp-1* RNAi completely blocked lifespan extension by bDR on lifespan but only partially blocked lifespan extension by the *daf-2* mutation at the ad lib concentration. Conversely, *daf-16* RNAi completely blocked lifespan extension by the *daf-2* mutation but only partially blocked lifespan extension by bDR (Figure 2D, Table S1). Furthermore, whereas at the ad lib bacterial concentration in liquid media the *daf-2* mutation increased lifespan by about 30% and optimum bacterial dilution increased lifespan by 65%, the *daf-2* mutation further increased lifespan at the optimum bacterial concentration by 40% (Figure 2E, Table S1), consistent with previous conclusions that lifespan extension by the *daf-2* pathway and by DR are mediated by additive mechanisms [19]. Nevertheless, *cbp-1* RNAi completely blocked the increase in lifespan by the *daf-2* mutation at the optimum bacterial concentration (Figure 2E, Table S1), suggesting that under conditions of bDR *cbp-1* may participate in lifespan extension by the *daf-2* mutation. Similarly, *cbp-1* RNAi completely blocked lifespan extension by the *glp-1* and *clk-1* mutations (Table S1), the latter observation consistent with a key role of electron chain transport complex I in mediating effects of DR on lifespan [14].

Although both *cbp-1* RNAi and *daf-16* RNAi slightly reduced lifespan under standard conditions, and to the same extent (as is also the case with *smk-1* RNAi [22] and *pha-4* RNAi [4], similar to *hsf-1* and *dve-1*/*satb-1* RNAi; see below), the effect on mortality was only manifested late in life, indicating that increased mortality by *cbp-1* RNAi is specific to the aging process. Furthermore, *cbp-1* RNAi did not attenuate lifespan extension by low temperature (40% extension in controls vs. 44% extension in the *cbp-1* RNAi group) (Figure 2F). Similarly, *cbp-1* RNAi had no effect on lifespan in *daf-16* hypomorphic (m26) worms (Figure 2G and Table S1), and combining both *cbp-1* and *daf-16* RNAi in wild-type N2 worms produced a similar reduction in lifespan as produced by either *cbp-1* or *daf-16* RNAi alone (Table S1). Taken together, these studies demonstrate that lifespan reduction by *cbp-1* RNAi is not non-specific but depends on context, such that increased mortality due to *cbp-1* RNAi depends on age and *daf-16*, is greatest under conditions of dietary restriction, and is minimal under ad lib fed conditions.

### *cbp-1* RNAi Specifically Accelerates Aging

**Figure 3:**
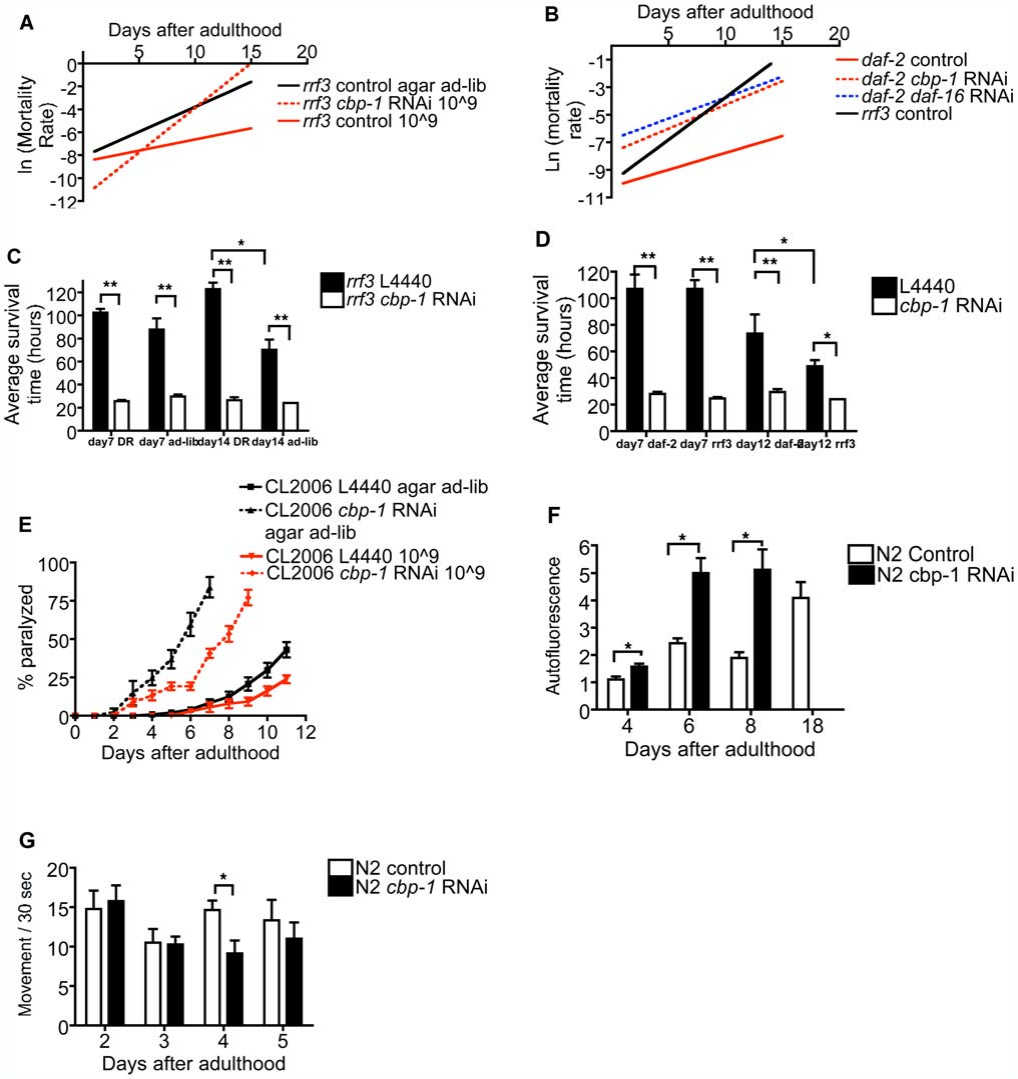
*Cbp-1* RNAi accelerates aging without producing non-specific toxicity. *cbp-1* RNAi reverses the reduction of mortality rate doubling time by (A) DR or (B) the *daf-2* mutation. The Gompertz variables G and A were calculated using non-linear regression on the Kaplan-Meier estimates of the survivorship curves. Line was generated from the calculated value of G (slope) and A (intercept). Mortality rate doubling time equals to Ln2 divided by G. *cbp-1* RNAi reverses resistance to 50 mM paraquat produced by (C) bDR or the (D) *daf-2* mutation. Data are quantified as average survival time in the paraquat solution and presented as mean ± SEM (*n* = 30/group, *p<0.01, **p<0.001). (E) *cbp-1* RNAi accelerates paralysis caused by age-dependent aggregation of transgenic human Aβ1-42. Data are quantified as average rate of paralysis per day and presented as mean ± SEM (*n* = 50–100/group). *cbp-1* RNAi (F) increases autofluorescence but (G) does not reduce activity. Data are presented as mean ± SEM (*n* = 6–10/group, *p<0.05).

To more directly assess the impact of *cbp-1* on the rate of aging, we quantified the effect of *cbp-1* RNAi on mortality rate doubling time, using the Gompertz model for mortality rate, h(t) = Ae^Gt^, in which G reflects age-dependent acceleration of mortality rate and thus the rate of aging, whereas A reflects the initial or constant component of mortality rate [23]. The effect of bDR and *cbp-1* RNAi were calculated using an improved and validated algorithm, based on a validated non-linear regression algorithm, as recently described [24]. Based on these analyses, bDR significantly reduced the rate of aging, an effect reversed by *cbp-1* RNAi (indeed, *cbp-1* RNAi significantly accelerated the rate of aging; Figure 3A and Table S2). By the same analysis, the *daf-2* mutation also significantly reduced the rate of aging, an effect similarly (though only partly) reversed both by *cbp-1* RNAi and *daf-16* RNAi (Figure 3B and Table S2). Another marker thought to reflect a fundamental process of aging, sensitivity to oxidative stress [25], as indicated by paraquat-induced mortality, was delayed by bDR and the *daf-2* mutation and strikingly enhanced by *cbp-1* RNAi (Figure 3C and 3D).

Mechanisms by which DR increases lifespan are of interest, but of even greater interest are mechanisms by which DR reduces the burden of age-related pathologies, including such disparate diseases as cancer and neurodegeneration [1]. We therefore assessed if *cbp-1* RNAi would block protective effects of bDR to delay the development of pathology in a transgenic model of Alzheimer's disease produced by expression of human *Aβ1-42* in *C. elegans* (CL2006) [7],[26]. Consistent with previous reports [7], bDR delayed the onset of paralysis produced by the human *Aβ1-42* transgene, and *cbp-1* RNAi greatly accelerated the onset of paralysis (Figure 3E). However, since *cbp-1* RNAi accelerated the onset of paralysis even in worms subjected to bDR (Figure 3E), and bDR significantly delayed the onset of paralysis even in the presence of *cbp-1* RNAi, the effect of *cbp-1* RNAi on *Aβ1-42*-dependent pathology may be independent of bDR.

To further assess if *cbp-1* RNAi reduces lifespan by accelerating aging or producing general sickness, we examined if *cbp-1* RNAi would increase autofluroescence, an indicator of aging, or reduce activity, an indicator of toxicity [27]. *cbp-1* RNAi significantly accelerated the accumulation of autofluorescence (Figure 3F) but had had no effect on activity (Figure 3G), the active portion of lifespan (65% for control versus 67% for *cbp-1* RNAi), egg laying (Figure S2), or pharyngeal pumping rate (Figure S3). Interestingly, however, *cbp-1* RNAi did reduce DiI staining in amphid neurons Figure S4).

### Inhibition of Three CBP-Interacting Factors Attenuates Lifespan Extension by bDR

**Figure 4:**
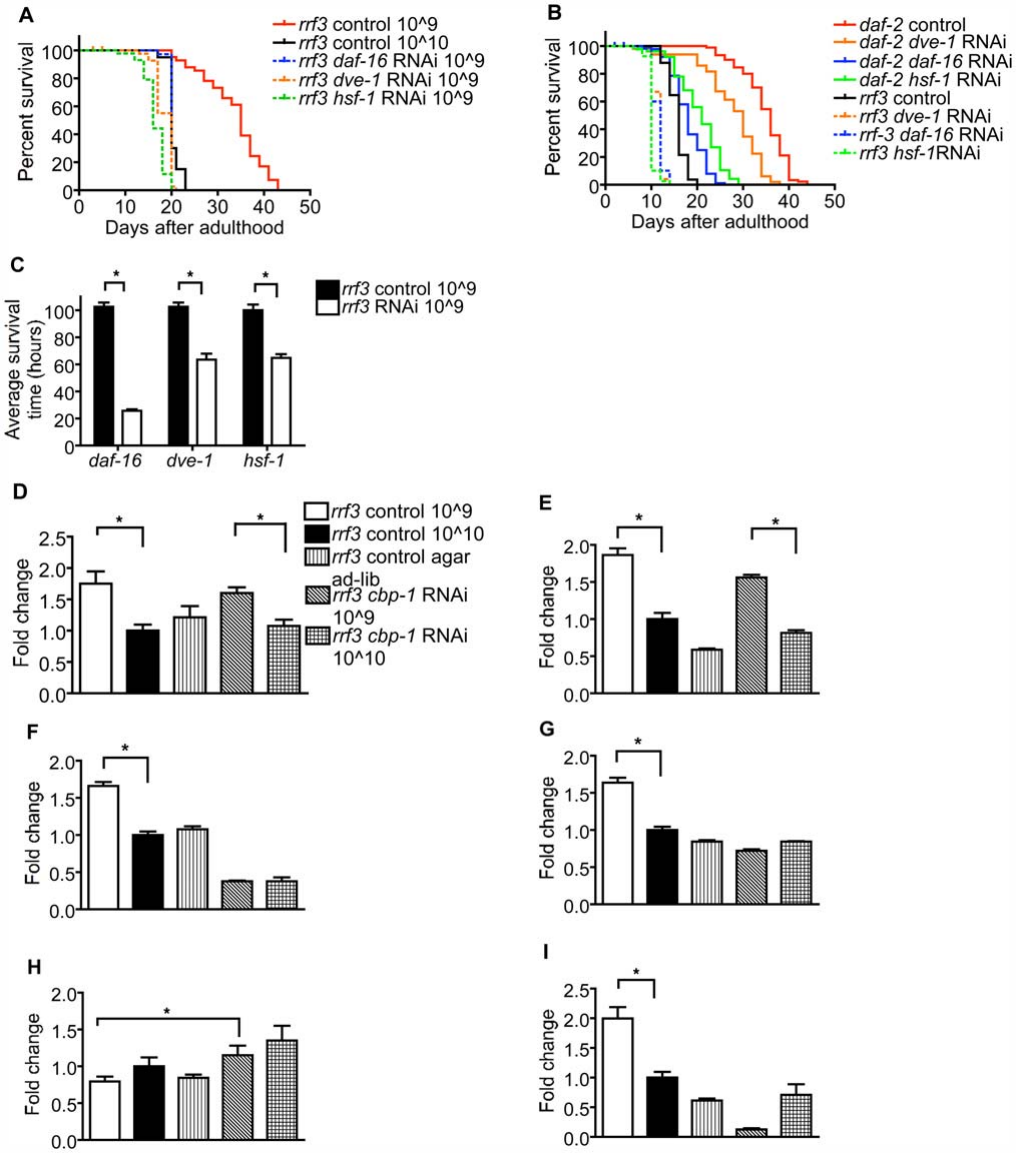
Inhibition of CBP-interacting factors attenuates lifespan extension by bDR. (A) *daf-16*, *hsf-1*, and *dve-1* RNAi attenuate lifespan extension by bDR. (B) *daf-16* and *hsf-1* but not *dve-1* RNAi blocks lifespan extension by the *daf-2* mutation. (C) *daf-16*, *hsf-1*, and *dve-1* RNAi decreases resistance to 50 mM paraquat. bDR induces expression of (D) *daf-16* and (E) *hsf-1*, but *cbp-1* RNAi does not block this induction. However, *cbp-1* RNAi blocks induction of (F), *sod-3*, (G) *sip-1*, *and* (I) W03F9.4/CPT-1 and inhibition of (H) Y71H10A.1 by bDR. Data are presented as mean ± SEM (*n* = 4–6/group, *p<0.05).

To discover genes mediating lifespan extension by bDR, we used RNAi to screen over 500 genes including factors that interact with CBP, are nutritionally regulated, or are otherwise reported to influence lifespan, for reduction of lifespan at the optimal bacterial concentration (see Methods). In this screen inhibition of only four genes reduced viability on adult day 20 at the optimum bacterial concentration: *cbp-1*, *daf-16*, *hsf-1*, and *dve-1* (Defective proVEntriculus in Drosophila), the *C. elegans* ortholog of *satb-1*. More detailed lifespan studies corroborated that *hsf-1*, *daf-16*, and *dve-1* attenuated lifespan extension by bDR, though *daf-16* RNAi and *dve-1* RNAi did not completely block lifespan extension by bDR (Table S1), whereas *cbp-1* and *hsf-1* RNAi did completely block lifespan extension by bDR (Table S1). Furthermore, *daf-16* RNAi also completely blocked lifespan extension by the *daf-2* mutation as previously reported [28], while *hsf-1*, *dve-1*, and *cbp-1* RNAi only partially blocked life extension by the *daf-2* mutation (Figure 4B). As with *cbp-1*, RNAi directed against all of these other three gene products attenuated protection against oxidative stress by bDR (Figure 4C).

### *Cbp-1* RNAi Blocks Effects of bDR on Expression of Genes Regulated by *daf-16* and *hsf-1* and Other Genes Regulating Metabolism

Mammalian FOXO3A [29], HSF-1 [30], and SATB-1 [31] all interact with CBP. In *C. elegans*, the interaction between DAF-16 and CBP-1 has been confirmed by yeast two-hybrid assays [29]. To assess possible interactions between *C. elegans cbp-1* and these CBP-binding gene products, we assessed the effect of *cbp-1 RNAi* on genes regulated by *daf-16* and *hsf-1*. *daf-16* and *hsf-1* were induced by bDR (Figure 4D and 4E), whereas, interestingly, *dve-1* expression was not induced (unpublished data). Though *cbp-1* RNAi did not block the induction of *daf-16* or *hsf-1* (Figure 4D and 4E) by bDR, it did block the induction of the DAF-16 target gene, *sod-3* [32], and the HSF-1 target gene, *sip-1* [28] by bDR (Figure 4F and 4G). *cbp-1* RNAi also blocked the induction of *sod-3* expression by *daf-2* RNAi (Figure S5). In contrast, *cbp-1* RNAi did not block increased Nile Red staining produced by the *daf-2* mutation, and in fact further enhanced Nile Red staining in *daf-2* mutant worms (Figure S7).

We also assessed the effects of bDR and *cbp-1* RNAi on 17 genes regulating glycolysis and beta-oxidation of lipids (based on their regulation by nutritional deprivation [33]), since the shift away from glycolysis toward beta-oxidation may contribute to protective effects of DR to increase lifespan and decrease disease burden [14]. As expected [14],[33], DR shifted metabolic balance away from glycolysis and toward beta-oxidation, as indicated by inhibited expression of Y71H10A.1, an ortholog of mammalian PFK, and F47G4.3, an ortholog of mammalian glycerophosphate dehydrogenase, and induced expression of ZK370.5, an ortholog of pyruvate dehydrogenase kinase (Figures 4H, S8, and S9), and W03F9.4, an ortholog of mammalian CPT1 (Figure 4I), as well as genes promoting peroxisome metabolism (unpublished data). As with genes regulated by *daf-16* and *hsf-1*, *cbp-1* RNAi blocked the effect of bDR on every metabolic gene whose expression was influenced by bDR (Figures 4D, 4E, 4F, 4G, 4H, 4I, S8, and S9).

### Age-Related Decrease in Histone Acetylation Level Is Delayed by bDR and Accelerated by *cbp-1* RNAi

**Figure 5:**
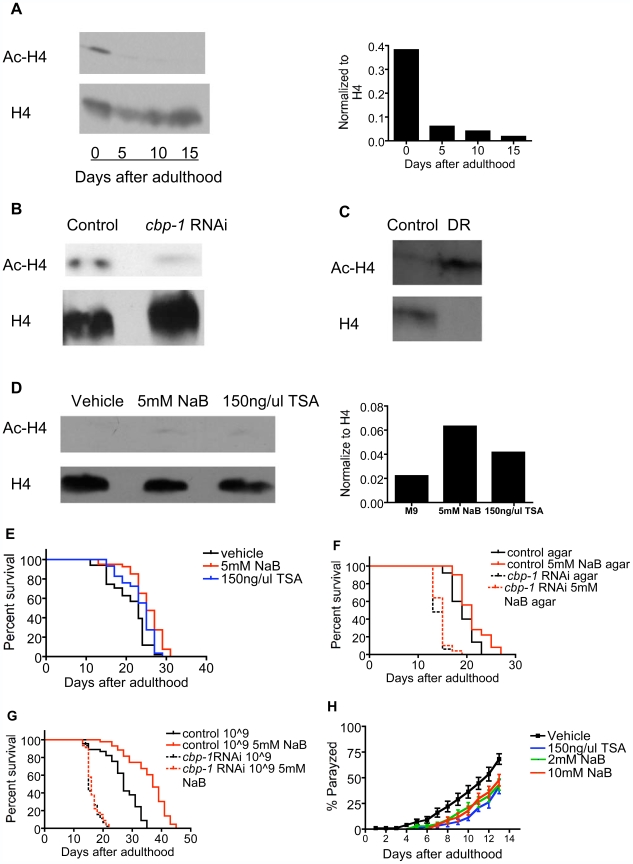
HDAC inhibitors increase histone acetylation and lifespan and delay pathology in a model of Alzheimer's disease. (A) Immunoblot for histone H4 and acetylated H4 (Lys5) in N2 worms during aging. Left: Immunoblot; Right: Densitometry (normalized to total H4). (B) Immunoblot for histone H4 and acetylated H4 (Lys5) in *rrf3* worms fed with control bacteria or bacteria expressing *cbp-1* dsRNA. (C) Immunoblot for histone H4 and acetylated H4 (Lys5) in N2 worms with bDR or control (ad lib) condition. (D) Immunoblot for histone H4 and acetylated H4 (Lys5) in the N2 worms treated with or without HDAC inhibitors (5 mM NaB and 150 ng/ul TSA). Left: Immunoblot; Right: Densitometry (normalized to total H4). (E) HDAC inhibitors (5 mM NaB and 150 ng/ul TSA) extend lifespan of N2 worms at 20°C. (F) Lifespan-extending effect of NaB is blocked by cbp-1 RNAi. N2 worms were treated with bacteria expressing cbp-1 dsRNA or control, and lifespan was measured in the presence or absence of 5 mM NaB at 20 C. (G) NaB further extended lifespan of bDR worms. N2 worms were treated with bacteria expressing *cbp-1* dsRNA or control at optimum bacterial dilution (10̂9 cells/ml) at 20 C. Lifespan was measured in the presence or absence of 5 mM NaB at 20C. *cbp-1 RNAi* completely blocked life extension by NaB and bDR. (H) NaB and TSA decrease Aβ1-42 toxicity as measured by rate of paralysis in Aβ1-42 transgenic worms (CL2006).

As a histone acetyltrasferase (HAT), CBP acetylates core histones and transcription factors [34], in particular histone H4 at lysine 5 [35]. To assess the relationship of HAT activity to lifespan, we examined acetylation of H4 Lys 5 during aging, and with bDR and *cbp-1* RNAi. Acetylation of H4 Lys 5, but not total H4, decreased with age (Figure 5A) and with *cbp-1* RNAi (Figure 5B) and increased with DR (Figure 5C).

### HDAC Inhibitors Increase Lifespan Dependent on *cbp-1* and Delay Aβ1-42-Dependent Paralysis

To assess if mimicking HAT activity chemically mimics protective effects of DR, we examined if two HDAC inhibitors, sodium butyrate (NaB) and Trichostatin A (TSA), would increase lifespan and reduce paralysis produced by the *Aβ1-42* transgene. Both HDAC inhibitors increased H4 Lys 5 acetylation (Figure 5D) and significantly increased lifespan at both ad lib and optimal bacterial concentrations, protective effects completely prevented by *cbp-1* RNAi (Figure 5E, 5F, and 5G, and Table S1). Similarly both HDAC inhibitors significantly delayed the onset of paralysis produced by the *Aβ1-42* transgene (Figure 5H).

## Discussion

In the present studies, we used two complementary screens to discover genes implicated in lifespan: an expression screen to discover genes whose hypothalamic expression predicts lifespan across five strains of mice, and an RNAi screen to discover genes whose inhibition blocks life extension by bDR. In the expression screen, hypothalamic expression of CBP and SATB-1, but not other genes previously implicated in influencing lifespan [2]–[9], correlated positively with lifespan. Since it is unlikely that polymorphisms in any single gene could account for such a large degree of variance in lifespan [36], we hypothesize that the differential hypothalamic expression of CBP and SATB-1 across strains is a quantitative trait reflecting many polymorphisms, which however sum to produce genetic variance in lifespan. It is not yet clear if the differential expression of CBP and SATB-1 that correlates with lifespan is confined to the hypothalamus, but *cbp-1* RNAi does produce dye-filling defects in all *C. elegans* amphid neurons (ASI, ADL, ASK, AWB, ASH, and ASJ). (Figure S4), consistent with the observation that a small number of neurons mediates lifespan extension by DR [3]. The identity of hypothalamic neurons whose expression of CBP predicts lifespan is therefore of particular interest.

Similarly, among genes whose expression was examined (including those previously implicated in mediating protective effects of DR), expression of only CBP, SATB-1, and HSF-1 decrease with age and diabetes. In the initial RNAi screen in *C. elegans*, of over 500 genes potentially associated with CBP or otherwise implicated in mediating effects of DR, only four genes were implicated in subsequent detailed analysis of lifespan extension by bDR: *cbp-1*, *daf-16*, *hsf-1*, and *dve-1*, the *C. elegans* ortholog of SATB-1. It should be noted, however, that whereas *cbp-1* and *hsf-1* RNAi completely block lifespan extension by bDR, *daf-16* and *dve-1* RNAi only partially block lifespan extension by bDR. Since molecular mechanisms mediating DR depend on the protocol of DR [8],[9], we further corroborated that *cbp-1* RNAi blocks lifespan extension by the *eat-2* mutation and liquid axenic media. To our knowledge *cbp-1* is the only gene implicated in lifespan extension by three different protocols of DR; it will therefore be of interest to determine if there are any protocols of DR in which life extension is not blocked by *cbp-1* RNAi [9].

Of the more than 500 genes screened in two distinct protocols, the gene most comprehensively implicated in mediating lifespan extension by bDR was *cbp*: expression of CBP accounts for the most variance in lifespan, decreases with age and diabetes, is induced by bDR and by the *daf-2* mutation, and *cbp-1* RNAi blocks lifespan extension by all three protocols of DR examined so far and partly attenuates lifespan extension by the *daf-2* mutation. Expression of SATB-1 accounts for almost as much variance of lifespan and decreases with age and diabetes but is not induced by bDR, and *dve-1* RNAi (ortholog of SATB-1) only partly blocks lifespan extension by bDR and the *daf-2* mutation. Expression of HSF-1 decreases with age and diabetes and is induced by bDR, and *hsf-1* RNAi completely blocks lifespan extension by bDR and the *daf-2* mutation but does not block lifespan extension by other protocols of DR [8], nor predicts lifespan. Expression of *daf-16* is induced by bDR, and *daf-16* RNAi completely blocks lifespan extension by the *daf-2* mutation, but only partially blocks lifespan extension by bDR and does not attenuate lifespan extension by other protocols of DR [8], nor does expression of FOX03A predict lifespan or decrease with age or diabetes. Nevertheless, since *cbp-1* RNAi greatly suppresses lifespan extension by the *daf-2* mutation (completely so under bDR conditions), we hypothesize that *cpb-1* also plays a role in mediating effects of the insulin/IGF1 signaling pathway on longevity, possibly through an interaction with *daf-16* [29].

The robustness with which *cbp-1* RNAi blocks lifespan extension by DR, and the observation that *cbp-1* RNAi slightly reduces lifespan in standard conditions, raises the key question of whether *cbp-1* RNAi reduces lifespan extension by DR by reversing the retardation of aging by DR or by increasing general sickness [27]. Several lines of evidence strongly support that *cbp-1* RNAi specifically acts by reversing effects of DR on the process of aging. First, it should be noted that *cbp-1*, *daf-16* and *hsf-1* RNAi, and *pha-4* hypomorphism [4] reduce lifespan in standard conditions to the same extent, and it is accepted that these latter genes influence lifespan by influencing the process of aging, not by increasing general sickness [4],[28]. As with *pha-4*, *daf-16*, and *hsf-1* RNAi, the effect of *cbp-1* RNAi to increase mortality in standard conditions is completely age-dependent, occurring only after midlife, and entails a doubling of the age-dependent acceleration of mortality rate without influencing initial mortality rate, inconsistent with age-independent general sickness. Furthermore, the effect of *cbp-1* RNAi on lifespan is highly context-dependent, reducing lifespan maximally in the context of DR (any of three protocols), partially in long-lived *daf-2* mutant worms, minimally in standard conditions, and not at all in *daf-16* mutant worms. Conversely, *daf-16* RNAi, which reduces lifespan in standard conditions to the same extent as does *cbp-1* RNAi, reduces lifespan maximally in long-lived *daf-2* mutant worms and only partly reduces lifespan in the context of DR [18],[4]. Thus the same logic that supports the conclusion that *daf-16* and *hsf-1* mediate lifespan extension by the *daf-2* mutation, and that *pha-4* mediates lifespan extension by bDR, would also appear to support the conclusion that *cbp-1* also mediates lifespan extension by bDR. Furthermore, *cbp-1* RNAi specifically blocks effects of bDR on parameters thought to reflect processes of aging, age-dependent acceleration of mortality rate, resistance to oxidative stress, and specific profile of metabolic and anti-oxidant gene expression. Similarly, *cbp-1* RNAi accelerates the onset of autofluorescence without reducing total activity, feeding activity, or egg-laying rate, thus meeting criteria previously described for processes specifically impinging on aging rather than general sickness [27]. Indeed, *cbp-1* has been implicated in a genome-wide RNAi screen as one of 41 genes potentially mediating life extension through the insulin/IGF1 signaling pathway by influencing the process of aging, not general sickness [27]. Of particular interest, bDR delays and *cbp-1* RNAi accelerates the onset of proteotoxicity, just as the *daf-2* mutation and DR delay, and inhibition of *daf-16* and *hsf-1* accelerates, the same toxicity [7],[28]. Taken together, these data strongly support that *cbp-1* mediates lifespan extension and reduction of age-related pathology by DR.

Mechanisms linking *cbp-1* expression to increased lifespan and reduced age-related pathology are therefore of great interest. It is suggestive that all genes implicated in the present screens are either CBP or CBP-binding co-activators [29]–[31]. Similarly, genes recently implicated in mediating lifespan extension by DR, *skn-1* and *pha-4*, also code for transcriptional factors that interact with CBP [37],[38]. Furthermore, transgenic overexpression of *cbp-1* did not significantly increase lifespan (Figure S6), suggesting that effects of CBP on lifespan requires increased expression of other factors. Uniquely among factors implicated in mediating lifespan extension by DR, RHEB-1 is not known to interact with CBP [6]. However, RHEB-1 mediates lifespan extension by intermittent fasting-induced longevity [6], which, in contrast to other protocols of DR (including those examined in the present studies), increases lifespan by reducing initial mortality rate, not by reducing age-related acceleration of mortality rate (Yen and Mobbs, unpublished data), a characteristic of lifespan regulation through chemosensory rather than caloric mechanisms [39]. We therefore hypothesize that CBP-interacting transcriptional complexes (whose composition may depend on DR protocol) mediate protective effects of reduced caloric intake, whereas other mechanisms mediate lifespan extension by chemosensory mechanisms.

How CBP-interacting transcriptional complexes would act to increase lifespan and reduce age-related pathologies remains to be fully elucidated. An important role for the protective effects of CBP HAT activity is implicated by the observation that HDAC inhibitors, which effectively amplify HAT activity, increase lifespan, an effect completely dependent on *cbp-1* (enhanced by bDR, which increases CBP activity, and completely blocked by *cpb-1* RNAi). Since HAT activity, by increasing histone acetylation, presumably enhances transcriptional flexibility, decreased *cbp* expression with age may contribute to age-related loss of adaptive capacity [40], in particular in learning and memory [41]. On the other hand, HDAC inhibitors increase lifespan less than does dietary restriction, suggesting that increased HAT activity only partly accounts for the mechanisms by which *cbp* induction by DR increases lifespan. Since *cbp-1* RNAi also blocks effects of bDR on expression of genes regulating metabolic pathways, we hypothesize that the protective effect of CBP is mediated through these metabolic effects, particularly through a shift from glycolysis to fatty acid oxidation [14], as previously reported [42] (SOD isoforms do not appear to play a role in lifespan extension with at least some protocols of DR [43]). Since glucose produces a transcriptional profile opposite that of DR [14], these studies also suggest that down-regulation of the same CBP transcriptional complex implicated here may play a role in the development of age-related diseases such as Huntington's disease [44], Alzheimer's disease, and diabetic complications.

## Material and Methods

### Mouse Gene Expression

All studies in mice were carried out with permission and in accordance with the Institutional Animal Care and Use Committee at Mount Sinai School of Medicine. Ten-week-old male mice (*n* = 10 mice per strain) were obtained from Jackson Laboratory. The strains, with average lifespans as reported [12],[13], were as follows (mean lifespan ± standard error in days): BALB/cByJ (648±20.6); A/J (662±20.4); C3H/HeJ (700±35); DBA/2J (722±30); C57Bl/6J (827±30). Animals were housed with free access to food and water under a 12:12 h light-dark cycle (lights on at 7:00 am). Mice were sacrificed, following a balanced design during the light period (10:00 am to 2:00 pm), by decapitation after a brief exposure to carbon dioxide. Hypothalamic tissue was quickly dissected, frozen on dry ice, and stored at −70°C until extraction of RNA. Primer and normalization sequence are given in the supplementary information.

### *C. elegans* and RNAi Strains

N2, *rrf3* (pk1426), *daf-2* (e1370), *daf-16* (m26), *eat-2* (ad1113), CL2006 [26], *glp-1 (e2141)*, *clk-1 (e2519)*, *mev-1 (kn-1)*, and sod-3::GFP transgenic (CF1553) worm strains were obtained from the *Caenorhabditis* Genetics Center (Minneapolis, MN). Worms were grown at 20°C unless described otherwise. *cbp-1*, *daf-16*, *hsf-1*, and *dev-1* dsRNA expressing bacterial strains were from the genomic RNAi library (produced by J. Ahringer at the Wellcome/CRC Institute). The bacterial strain containing empty vector L4440 was used as the control. RNAi was carried out following standard bacterial feeding methods [45]. All RNAi experiments were performed only in adult worms.

### Protocols for Dietary Restriction

#### Bacterial dilution

Bacteria concentration was obtained by measuring OD600. The relation between OD600 and bacteria concentration was determined by colony formation assay (BC = OD600/0.0121×10̂7 cells/ml). After obtaining concentration of each culture, control or RNAi bacteria was re-suspended in S basal media supplemented with flurodeoxyuridine (FUDR) (0.1 g/L), ampicillin (0.1 g/L), and IPTG (0.24 g/L) to make concentrations of 10̂8, 10̂9, and 10̂10 cells/ml. Lifespan study was performed at each dilution.

#### Axenic media

The liquid axenic media [16] consisted of 3% Soy Peptone, 3% Yeast Extract, 0.05% Hemoglobin, and 94% water, supplemented with FUDR (0.1 g/L) and ampicillin (0.1 g/L). Adult worms were grown on solid plates with appropriate RNAi or control constructs for 5 d, then transferred to liquid axenic media, or to control agar ad-lib plates with no RNAi, and scored every 2 d.

### Lifespan Analysis

Eggs were collected by standard hypochlorite treatment of gravid worms. Worms were grown on 100 mm NGM plates until the L4/early adult stage was reached. The worms were then transferred to 35 mm NGM plates supplemented with 5-FUDR (0.1 g/ml) with 10 worms on each plate. The worms were scored every 2 d and were categorized as dead when they failed to respond to gentle prodding from a platinum wire. For bacterial dilution, bacteria containing dsRNA or empty vector were suspended in S basal medium supplemented with FUDR (0.1 g/L), ampicillin (0.1 g/L), and IPTG (0.24 g/L) at the concentrations of 10̂8, 10̂9, or 10̂10 cells/ml. The worms were transferred to 24 well plates with 15 worms per well and scored every 2 d. For studies in liquid axenic media, adult worms were grown on RNAi or control solid media plates for 5 d, then transferred to liquid axenic media afterward and scored every 2 d.

### RNAi Screening

RNAi screening was carried out using 24 well plates. *E. coli* expressing various dsRNAs were prepared at the concentration of 10̂9 cells/ml. The synchronized *rrf3* worms just reaching adulthood were washed off agar plates and suspended in M9 buffer. Around 25 worms were pipetted into each *E. coli* solution. On day 15 and day 20, the number of dead worms was counted. Genes whose inhibition by RNAi reduced the number of live worms at day 20 (according to visual inspection) were further assessed in more detailed lifespan assays; only four genes out of more than 500 tested met this initial criterion, and lifespan reducing effects were corroborated for all four.

### Paraquat Assay

Thirty worms from each experimental group were transferred into 50 ul of a 50 mM paraquat solution with 10 worms per well in a 96 well plate. Worms were scored every 24 h and were categorized as dead when they failed to respond to gentle prodding from a platinum wire.

### Paralysis Assay

The paralysis assay followed the method described by Cohen et al [46]. Briefly, 50 worms were placed on plates or in wells (10 worms per plate or well). The worms were tested daily by tapping the head with a platinum wire. Worms that moved their heads but failed to move their bodies were categorized as paralyzed. To avoid scoring of dead animals as paralyzed, paralysis assay was terminated at day 12 of adulthood unless described otherwise.

### RNA Isolation and Quantitative Real-Time PCR (q-PCR)

Total RNA was isolated from synchronized populations at day 7 of adult worms. Total RNA was extracted using TRIzol reagent (Invitrogen, 15596-018). 1 ug total RNA from each sample was converted into cDNA with 100 pg utilized for each individual q-PCR assay in a 40 cycle, three-step PCR reaction using the ABI Prism 7900 thermocycler with 384-well thermal cycling block module and robot arm. Amplification was performed in 20 mM Tris pH 8.4, 3 mM MgCl2, 200 mM dNTPs, 0.5% SYBR green (Molecular Probes), 200 uM of each primer, and 0.25 U platinum Taq (Life Technologies). Quantification was completed using SDS2.1 software (Applied Biosystems).

Mouse primer sequences:

CBP (NM_001025432)

forward: 5′-ATGGCTGCTCCAATGGGACAAC-3′

backward: 5′-GCACCTGGTTACTAAGGGATG-3′

SATB-1 (NM_001163630)

forward: 5′-GCCAATGAATCTTCGGAAACTTGC-3′

backward: 5′-TACCAAACAGTCCTTGACGCTTC-3′

HSF-1 (NM_019740)

forward: 5′-GAGACAAAACAGTTGGGTAGTCCAG-3′

backward: 5′-TGGGGTAAAGGCAGCAGGTATG-3′

FOXO3A (NM_019740)

forward: 5′-AAGGGGAAATGGGCAAAGC-3′

backward: 5′-cgtgggagtctcaaaggtgtcaag-3′

Normalization gene-mouse tubulin alpha 1 (BC002219)

forward: 5′-TGCCTTTGTGCACTGGTATG-3′

reverse: 5′-CTGGAGCAGTTTGACGACAC-3′

*C. elegans* primer sequences:

*cbp-1* (R10E11.1)

forward: 5′-ACACCAACAGCCACCACCTTTC-3′

backward: 5′-TTCCCGCATCCTAAGCCAAG-3′

*daf-16* (R13H8.1)

forward: 5′-ACGAGTTGAACAGTGTCCGTGG-3′

backward: 5′-GGCAGTGGAGATGAGTTGGATG-3′

*hsf-1* (Y53C10A.12)

forward: 5′-CCGTTGGATGATGATGAAGAAGG-3′

backward: 5′-CAAGTGGCAAGTAGTTTGGGTCC-3′

*sod-3* (C08A9.1)

forward: 5′-CAACTTGGCTAAGGATGGTGGAG-3′

backward: 5′-CTTGAACCGCAATAGTGATGTCAG-3′

*sip-1* (F43D9.4)

forward: 5′-ACAACATCGTGCCACAACAGC-3′

backward: 5′-CGTCCTTTGGAAGAGTGAACTGG-3′

Y71H10A.1

forward: 5′-GAAGAGCCATTCACCGTTCAAG-3′

backward: 5′-CCCACTCATTTCTGACAACCAAG-3′

W03F9.4

forward: 5′-GAACTGCGATGGCTGGAAAAG-3′

backward: 5′-TTGAGGGGTCTGGCTTGTGGATAG-3′

F47G4.3

forward: 5′-TGTTCTGATGGGAGCCAACTTG-3′

backward: 5′-CGGATGATAGCGGATTTCACG-3′

ZK370.5

forward: 5′-ACGCTCGGTATTTCCTTGG-3′

backward: 5′-GCTACTTGTGGTCCCATTGTC-3′

Normalization gene F23B2.13 [47]

forward: 5′-CGCCGAAAATGAAATCAAAC-3′

backward: 5′-GGGCGTCGTACACCATCA-3′

### Immunoblot Protocol

50–100 worms from each experimental group were boiled in 20 ul 2X sample buffer for 20 min. After 5 min chilling on ice, the whole sample was loaded onto SDS-PAGE for immunoblotting. Equal loading was assured as the same number of worms was used in each experiment. Antibodies CBP (C-1): Sc-7300 and DAF-16 (ce-300): Sc-33738 were purchased from Santa Cruz Biotechnology Inc. Anti-Histone H4 (#07-108) and anti-H4 (Lys5) (07-327) were purchased from Upstate. Immunoblots were carried out following standard procedures (Dilutions: anti-CBP antibody 1∶200, anti-DAF-16 antibody 1∶500, anti-H4 antibody 1∶3000, and anti-H4 (Lys5) antibody 1∶2000).

### Drug Treatments

TSA (#19-138) was purchased from Upstate and prepared at a concentration of 150 ng/ul. Sodium butyrate (NaB) was obtained from Sigma and prepared at the concentration from 2, 5, or 10 mM. The drug solution was added onto seeded plates and distributed evenly on the surface of the agar. Worms just reaching adulthood were transferred to the plates with or without drugs and lifespan or paralysis assay was conducted as described above.

### Aging Phenotype Studies

To assess autofluorescence, 6–10 N2 worms from each experiment group were transferred onto a slide and images were taken by fluorescence microscope. Green fluorescence intensity of each worm was measured using software image-J. For movement studies, adult worms that only moved head or tail but failed to move the body upon prodding were considered as immobile and the number of immobile worms was counted daily. The ability to swim in water was quantified as the number of full movement back and forth within 30 s. To assess egg laying, two worms were placed onto a seeded NGM plate and were transferred daily to fresh plates. The number of eggs left on the plate was counted daily. Ten plates were used for each experiment group.

### Statistical Analysis

Analysis of lifespan began with an estimation of the survival curve using the Kaplan-Meier product limit estimate of the survivorship function. The Gompertz variables G and A were calculated using non-linear regression on the Kaplan-Meier estimates of the survivorship curves; although another parameter, M_0_, can be included in such an analysis, we have found that this parameter is essentially 0 when included in the analysis [24] and is therefore not included. After the non-linear regression determined the values for G, the extra sum-of-squares *F*-test was used to assess significant effects on G values. The analysis was completed using Prism 4 software. For survival studies, statistical significance was determined using Log-rank test. For paraquat studies, paralysis assays, and q-PCR, statistical significance was determined using *t*-tests. For mouse gene expression studies, linear regression was used to determine the correlation between mRNA levels and average lifespan across the five strains of mice.

## Supporting Information

Figure S1 **Bacterial dilution extended lifespan in** ***C. elegans*** **.** *E. coli* was prepared at 5*10̂7, 10̂8, 2*10̂8, 5*10̂8, 10̂9, 2*10̂9, 5*10̂9, and 10̂10 cells/ml and average lifespan of the *rrf3* worms was measured.
(2.58 MB TIF)

Figure S2 ***cbp-1*** **RNAi does not affect the p-pumping rate in N2 worms.** Data are quantified as the number of pharyngeal pumping within 15 s and presented as mean ± SEM (*n* = 6–10/group).
(2.77 MB TIF)

Figure S3 ***cbp-1*** **RNAi does not affect egg laying in N2 worms.** Data are quantified as the number of eggs per day per two worms and presented as mean ± SEM (*n* = 10/group).
(2.67 MB TIF)

Figure S4 ***cbp-1*** **RNAi produces DiI staining defect.** *rrf3* worms were grown at optimum bacterial concentration and fed with either control bacteria (L4440) or bacteria expressing *cbp-1* dsRNA for 7 d. The neuron-specific dye DiI was added into the culture and worms were stained for 2 h. Images were taken by confocal microscope using a 40X objective lens. Red staining: amphid neurons.
(6.62 MB TIF)

Figure S5 ***cbp-1*** **and** ***daf-16*** **RNAi similarly block the induction of** ***sod-3*** **by the** ***daf-2*** **mutation.** *sod-3*::GFP transgenic worms (CF1553) were fed with *daf-2* dsRNA alone (A), *daf-2* and *cbp-1* dsRNA (B), *daf-2* and *daf-16* dsRNA (C), or control L4440 empty vector (D) at 20 C, and photographed by fluorescence microscope. RNAi dilution effect was controlled by 1∶1 dilution of *daf-2* RNAi with L4440. (E) *cbp-1* RNAi and *daf-16* RNAi equally decreased *sod-3* expression. Average GFP fluorescence was quantified using software imageJ and difference between groups was determined by student's *t*-test (*p<0.05, *n* = 5–7/group).
(4.72 MB TIF)

Figure S6 **Overexpression of** ***cbp-1*** **does not extend lifespan in N2 worms at 25°C.** Transgenic worms were generated by co-microinjecting PCR product *cbp-1::cbp-1* and *rol-6* plasmid (pRF4) into N2 worms. Two independent transgenic lines were maintained and used in lifespan assay. Control worms were generated by injecting *rol-6* plasmid alone. No lifespan extension was observed by *cbp-1* overexpression (*p* = 0.11 line 1 versus control, *p* = 0.14 line 2 versus control).
(2.65 MB TIF)

Figure S7 ***cbp-1*** **RNAi increases, rather than reverses, increased Nile Red staining produced by the** ***daf-2*** **mutation.** *daf-2* mutant worms were fed with control bacteria or bacteria expressing *cbp-1* dsRNA for 5 d at 25°C. Worms were stained with Nile Red dye and fluorescence was quantified software ImageJ. Data are presented as mean ± SEM (*n* = 5/group, *p*<0.05).
(2.58 MB TIF)

Figure S8 ***cbp-1*** **RNAi blocks the inhibition of F47G4.3, an ortholog of mammalian glycerophosphate dehydrogenase, by bDR.** Data are presented as mean ± SEM (*p<0.05, *n* = 4–6/group).
(2.81 MB TIF)

Figure S9 ***cbp-1*** **RNAi blocks the induction of ZK370.5, an ortholog of mammalian pyruvate dehydrogenase kinase, by bDR.** Data are presented as mean ± SEM (*p<0.05, *n* = 4–6/group).
(2.74 MB TIF)

Table S1 **Summary of lifespan assays.**
(0.18 MB DOC)

Table S2 **Gompertz values for lifespan assays.**
(0.04 MB DOC)

## Abbreviations

bDR: DR produced by dilution of bacteria in liquid media to produced optimum lifespan
CBP: CREB-binding protein
CITED-1: CBP/p300-interacting transactivator with Glu/Asp-rich carboxy-terminal domain 1
DR: dietary restriction
FUDR: flurodeoxyuridine
HAT: histone acetyltransferase
HDAC: histone deacetylase
q-PCR: quantitative real-time PCR
RNAi: RNA interference
SATB-1: special AT-rich sequence binding protein 1
TSA: Trichostatin A

## References

[1] Mobbs C. V, Hof P. R (2007). Mechanisms of dietary restriction in aging and disease..

[2] Lin S. J, Defossez P. A, Guarente L (2000). Requirement of NAD and SIR2 for life-span extension by calorie restriction in Saccharomyces cerevisiae.. Science.

[3] Bishop N. A, Guarente L (2007). Two neurons mediate diet-restriction-induced longevity in C. elegans.. Nature.

[4] Panowski S. H, Wolff S, Aguilaniu H, Durieux J, Dillin A (2007). PHA-4/Foxa mediates diet-restriction-induced longevity of C. elegans.. Nature.

[5] Greer E. L, Dowlatshahi D, Banko M. R, Villen J, Hoang K (2007). An AMPK-FOXO pathway mediates longevity induced by a novel method of dietary restriction in C. elegans.. Curr Biol.

[6] Honjoh S, Yamamoto T, Uno M, Nishida E (2009). Signalling through RHEB-1 mediates intermittent fasting-induced longevity in C. elegans.. Nature.

[7] Steinkraus K. A, Smith E. D, Davis C, Carr D, Pendergrass W. R (2008). Dietary restriction suppresses proteotoxicity and enhances longevity by an hsf-1-dependent mechanism in Caenorhabditis elegans.. Aging Cell.

[8] Mair W, Panowski S. H, Shaw R. J, Dillin A (2009). Optimizing dietary restriction for genetic epistasis analysis and gene discovery in C. elegans.. PLoS One.

[9] Greer E. L, Brunet A (2009). Different dietary restriction regimens extend lifespan by both independent and overlapping genetic pathways in C. elegans.. Aging Cell.

[10] Mobbs C. V, Bray G. A, Atkinson R. L, Bartke A, Finch C. E (2001). Neuroendocrine and pharmacological manipulations to assess how caloric restriction increases life span.. J Gerontol A Biol Sci Med Sci.

[11] Mastaitis J. W, Cheng H, Sealfon S. C, Mobbs C. V (2005). Acute induction of gene expression in brain and liver by insulin-induced hypoglycemia.. Diabetes 54.

[12] Goodrick C. L (1975). Life-span and the inheritance of longevity of inbred mice.. J Gerontol.

[13] Ingram D, Reynolds M, Les E (1982). The relationship between genotype, sex, body weight, and growth parameters to lifespan in inbred and hybrid mice.. Mech Ageing Dev.

[14] Mobbs C. V, Isoda F, Mastaitis J, Zhang M, Yang X. J (2007). Secrets of the lac operon: glucose hysteresis as a mechanism in aging, dietary restriction, and disease..

[15] Shi Y, Mello C (1998). A CBP/p300 homolog specifies multiple differentiation pathways in Caenorhabditis elegans.. Genes Dev.

[16] Houthoofd K, Braeckman B. P, Lenaerts I, Brys K, De Vreese A (2002). Axenic growth up-regulates mass-specific metabolic rate, stress resistance, and extends life span in Caenorhabditis elegans.. Exp Gerontol.

[17] Lee G. D, Wilson M. A, Zhu M, Wolkow C. A, de Cabo R (2006). Dietary deprivation extends lifespan in Caenorhabditis elegans.. Aging Cell.

[18] Lakowski B, Hekimi S (1998). The genetics of caloric restriction in Caenorhabditis elegans.. Proc Natl Acad Sci U S A.

[19] Houthoofd K, Braeckman B. P, Johnson T. E, Vanfleteren J. R (2003). Life extension via dietary restriction is independent of the Ins/IGF-1 signalling pathway in Caenorhabditis elegans.. Exp Gerontol.

[20] Simmer F, Tijsterman M, Parrish S, Koushika S. P, Nonet M L (2002). Loss of the putative RNA-directed RNA polymerase RRF-3 makes C. elegans hypersensitive to RNAi.. Curr Biol.

[21] Kenyon C, Chang J, Gensch E, Rudner A, Tabtiang R (1993). A C. elegans mutant that lives twice as long as wild type.. Nature.

[22] Wolff S, Ma H, Burch D, Maciel G. A, Hunter T (2006). SMK-1, an essential regulator of DAF-16-mediated longevity.. Cell.

[23] Johnson T. E (1990). Increased life-span of age-1 mutants in Caenorhabditis elegans and lower Gompertz rate of aging.. Science.

[24] Yen K, Steinsaltz D, Mobbs C. V (2008). Validated analysis of mortality rates demonstrates distinct genetic mechanisms that influence lifespan.. Exp Gerontol.

[25] Sohal R. S, Weindruch R (1996). Oxidative stress, caloric restriction, and aging.. Science.

[26] Link C. D (1995). Expression of human beta-amyloid peptide in transgenic Caenorhabditis elegans.. Proc Natl Acad Sci U S A.

[27] Samuelson A. V, Carr C. E, Ruvkun G (2007). Gene activities that mediate increased life span of C. elegans insulin-like signaling mutants.. Genes Dev.

[28] Hsu A. L, Murphy C. T, Kenyon C (2003). Regulation of aging and age-related disease by DAF-16 and heat-shock factor.. Science.

[29] Nasrin N, Ogg S, Cahill C. M, Biggs W, Nui S (2000). DAF-16 recruits the CREB-binding protein coactivator complex to the insulin-like growth factor binding protein 1 promoter in HepG2 cells.. Proc Natl Acad Sci U S A.

[30] Hong S, Kim S. H, Heo M. A, Choi Y. H, Park M. J (2004). Coactivator ASC-2 mediates heat shock factor 1-mediated transactivation dependent on heat shock.. FEBS Lett.

[31] Wen J, Huang S, Rogers H, Dickinson L. A, Kohwi-Shigematsu T (2005). SATB1 family protein expressed during early erythroid differentiation modifies globin gene expression.. Blood.

[32] Oh S. W, Mukhopadhyay A, Dixit B. L, Raha T, Green M. R (2006). Identification of direct DAF-16 targets controlling longevity, metabolism and diapause by chromatin immunoprecipitation.. Nat Genet.

[33] Mobbs C. V, Yen K, Mastaitis J, Nguyen H, Watson E (2004). Mining microarrays for metabolic meaning: nutritional regulation of hypothalamic gene expression.. Neurochem Res.

[34] Bannister A. J, Kouzarides T (1996). The CBP co-activator is a histone acetyltransferase.. Nature.

[35] McManus K. J, Hendzel M. J (2001). CBP, a transcriptional coactivator and acetyltransferase.. Biochem Cell Biol.

[36] Mobbs C. V, Rowe J. W (2001). Nature vs nurture in the aging brain..

[37] Hung H. L, Kim A. Y, Hong W, Rakowski C, Blobel G. A (2001). Stimulation of NF-E2 DNA binding by CREB-binding protein (CBP)-mediated acetylation.. J Biol Chem.

[38] Rausa F. M, Tan Y, Costa R. H (2003). Association between hepatocyte nuclear factor 6 (HNF-6) and FoxA2 DNA binding domains stimulates FoxA2 transcriptional activity but inhibits HNF-6 DNA binding.. Mol Cell Biol.

[39] Yen K, Mobbs C. V (2008). Chemosensory and caloric mechanisms influence distinct components of mortality rate.. Exp Gerontol.

[40] Mobbs C. V (2001). Adaptive capacity during aging..

[41] Mouravlev A, Dunning J, Young D, During M. J (2006). Somatic gene transfer of cAMP response element-binding protein attenuates memory impairment in aging rats.. Proc Natl Acad Sci U S A.

[42] Kwon H. S, Huang B, Ho Jeoung N, Wu P, Steussy C. N (2006). Retinoic acids and trichostatin A (TSA), a histone deacetylase inhibitor, induce human pyruvate dehydrogenase kinase 4 (PDK4) gene expression.. Biochim Biophys Acta.

[43] Yen K, Patel H. B, Lublin A. L, Mobbs C. V (2009). SOD isoforms play no role in lifespan in ad lib or dietary restricted conditions, but mutational inactivation of SOD-1 reduces life extension by cold.. Mech Ageing Dev.

[44] Nucifora F. C, Sasaki M, Peters M F, Huang H, Cooper J K (2001). Interference by huntingtin and atrophin-1 with cbp-mediated transcription leading to cellular toxicity.. Science.

[45] Wang J, Barr M. M (2005). RNA interference in Caenorhabditis elegans.. Methods Enzymol.

[46] Cohen E, Bieschke J, Perciavalle R. M, Kelly J. W, Dillin A (2006). Opposing activities protect against age-onset proteotoxicity.. Science.

[47] Fonte V, Kapulkin V, Taft A, Fluet A, Friedman D (2002). Interaction of intracellular beta amyloid peptide with chaperone proteins.. Proc Natl Acad Sci U S A.

