# Supplementary figures and images for: Role of CBP and SATB-1 in Aging, Dietary Restriction, and Insulin-Like Signaling
Source: PLoS Biol. 2009 Nov 17;7(11):e1000245. doi: 10.1371/journal.pbio.1000245 (PMC2774267; doi:10.1371/journal.pbio.1000245)

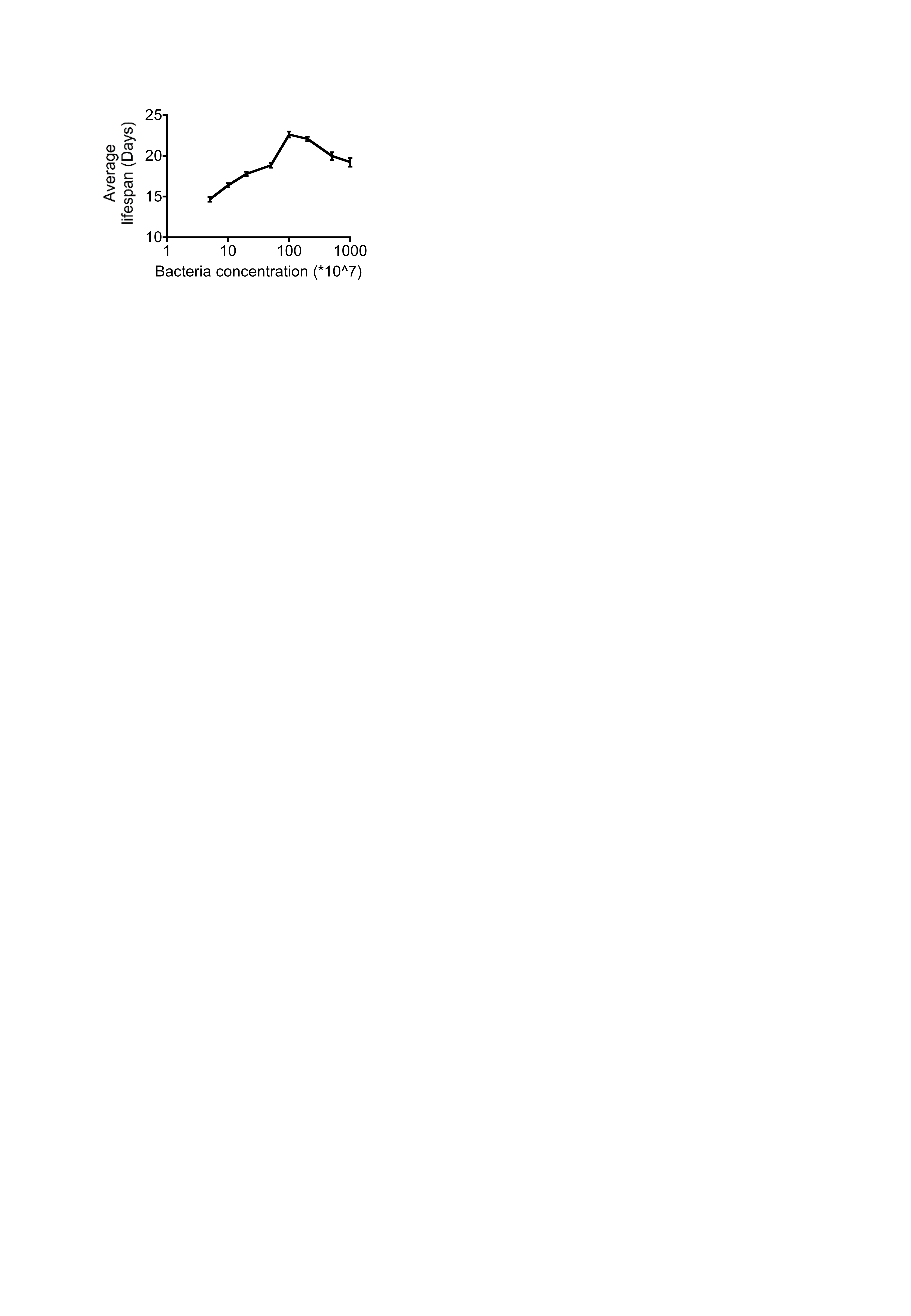

Supplement: Figure S1 — Bacterial dilution extended lifespan in C. elegans . E. coli was prepared at 5*10̂7, 10̂8, 2*10̂8, 5*10̂8, 10̂9, 2*10̂9, 5*10̂9, and 10̂10 cells/ml and average lifespan of the rrf3 worms was measured. (2.58 MB TIF) [file pbio.1000245.s001.tif]

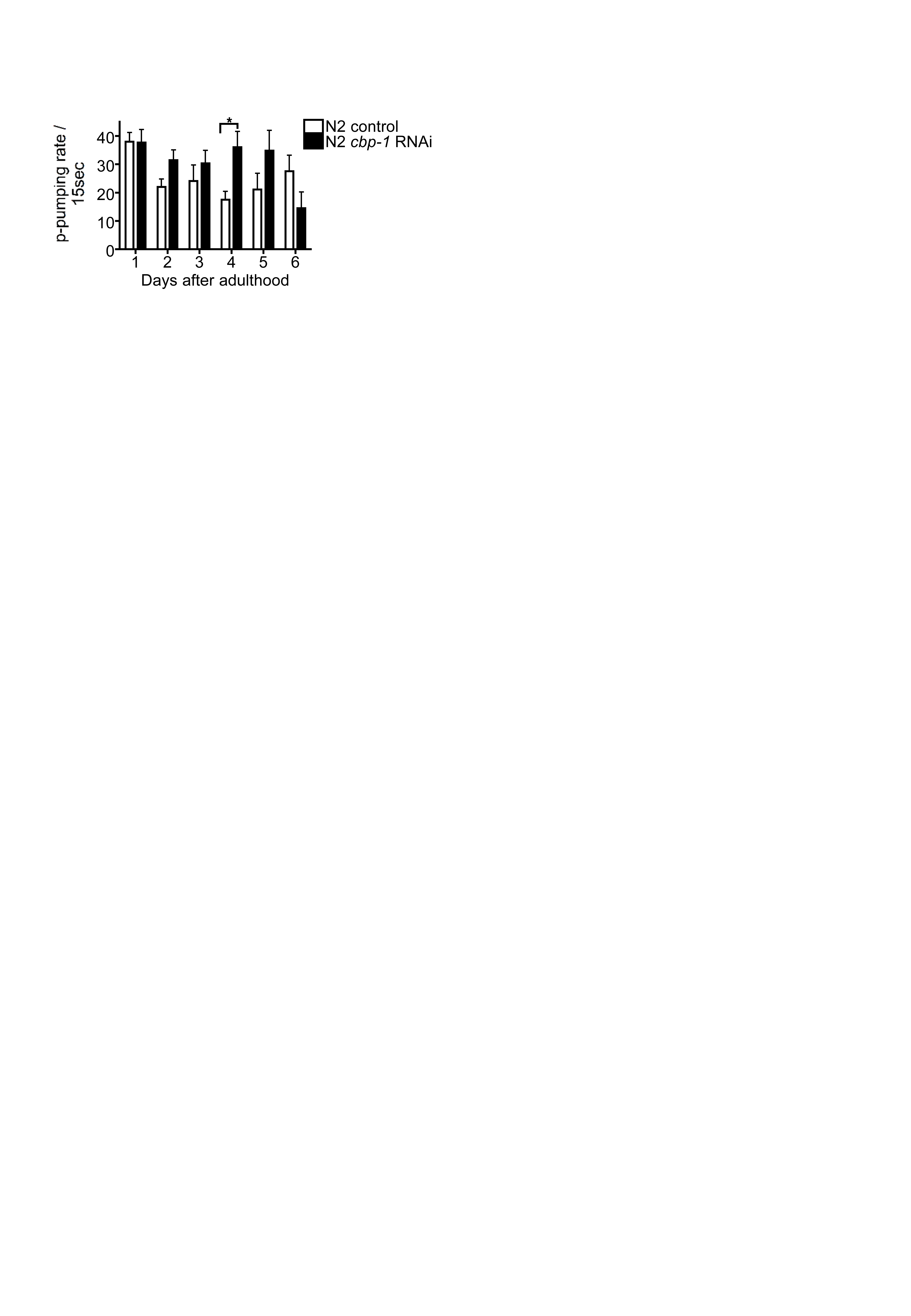

Supplement: Figure S2 — cbp-1 RNAi does not affect the p-pumping rate in N2 worms. Data are quantified as the number of pharyngeal pumping within 15 s and presented as mean ± SEM (n = 6–10/group). (2.77 MB TIF) [file pbio.1000245.s002.tif]

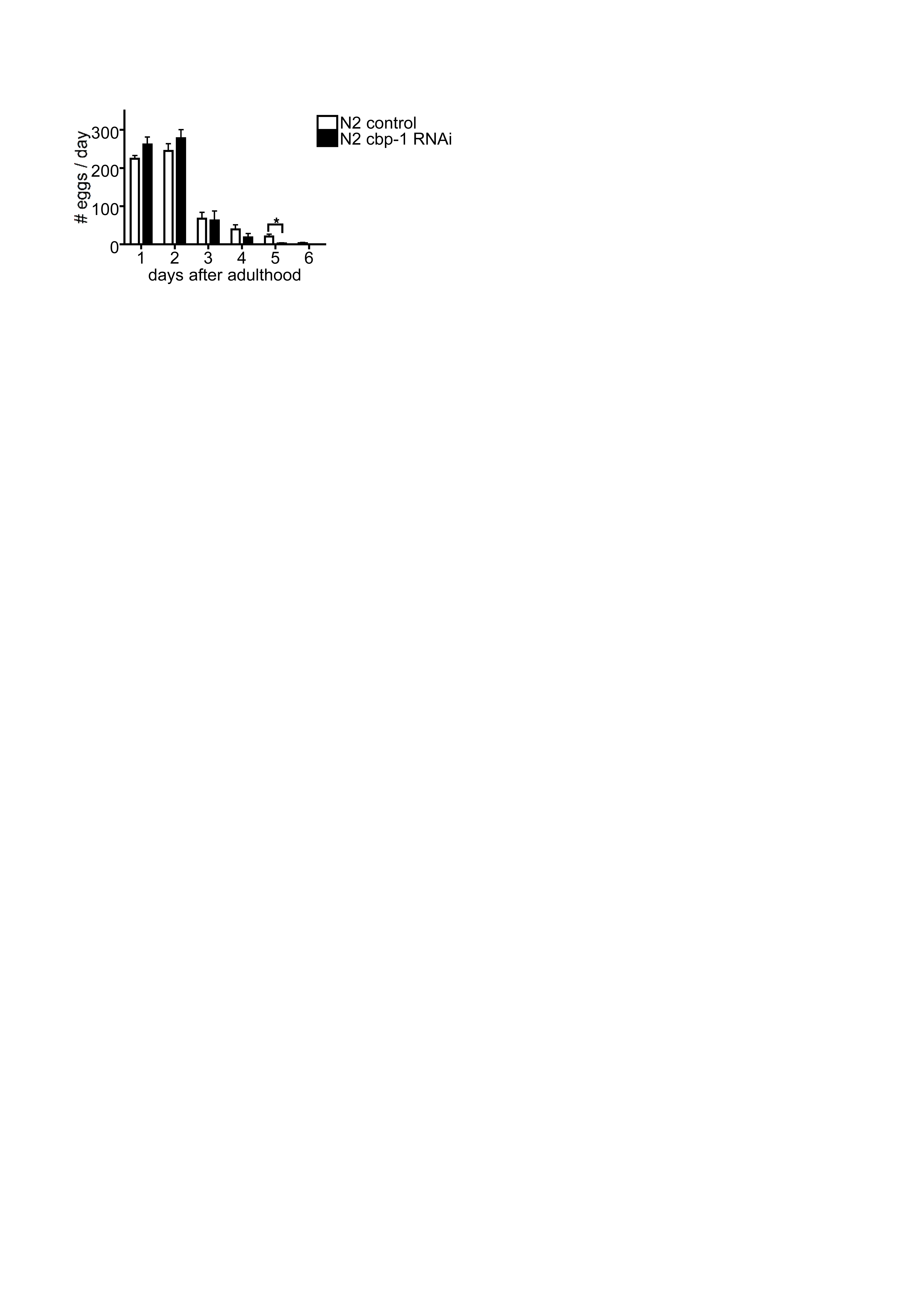

Supplement: Figure S3 — cbp-1 RNAi does not affect egg laying in N2 worms. Data are quantified as the number of eggs per day per two worms and presented as mean ± SEM (n = 10/group). (2.67 MB TIF) [file pbio.1000245.s003.tif]

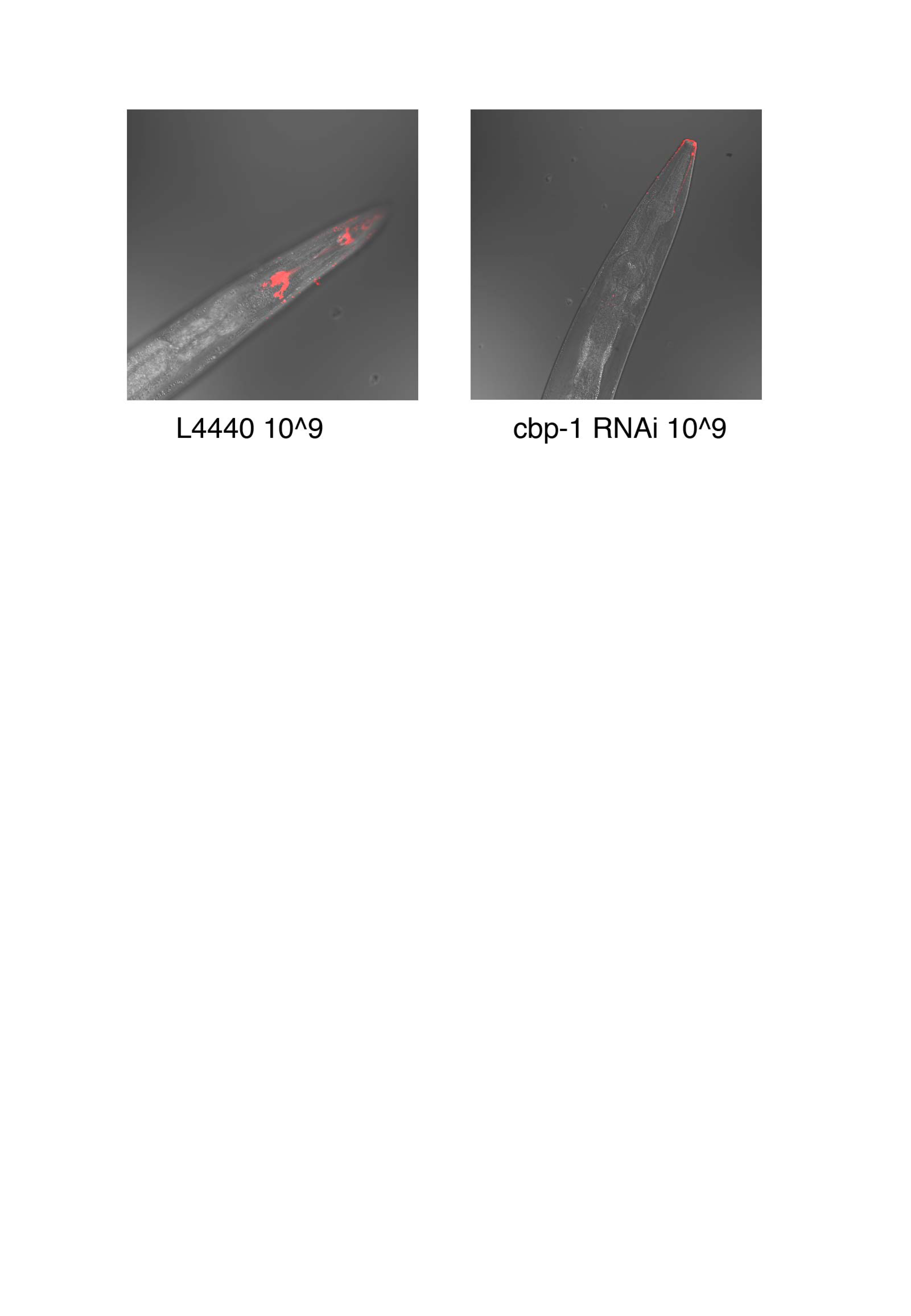

Supplement: Figure S4 — cbp-1 RNAi produces DiI staining defect. rrf3 worms were grown at optimum bacterial concentration and fed with either control bacteria (L4440) or bacteria expressing cbp-1 dsRNA for 7 d. The neuron-specific dye DiI was added into the culture and worms were stained for 2 h. Images were taken by confocal microscope using a 40X objective lens. Red staining: amphid neurons. (6.62 MB TIF) [file pbio.1000245.s004.tif]

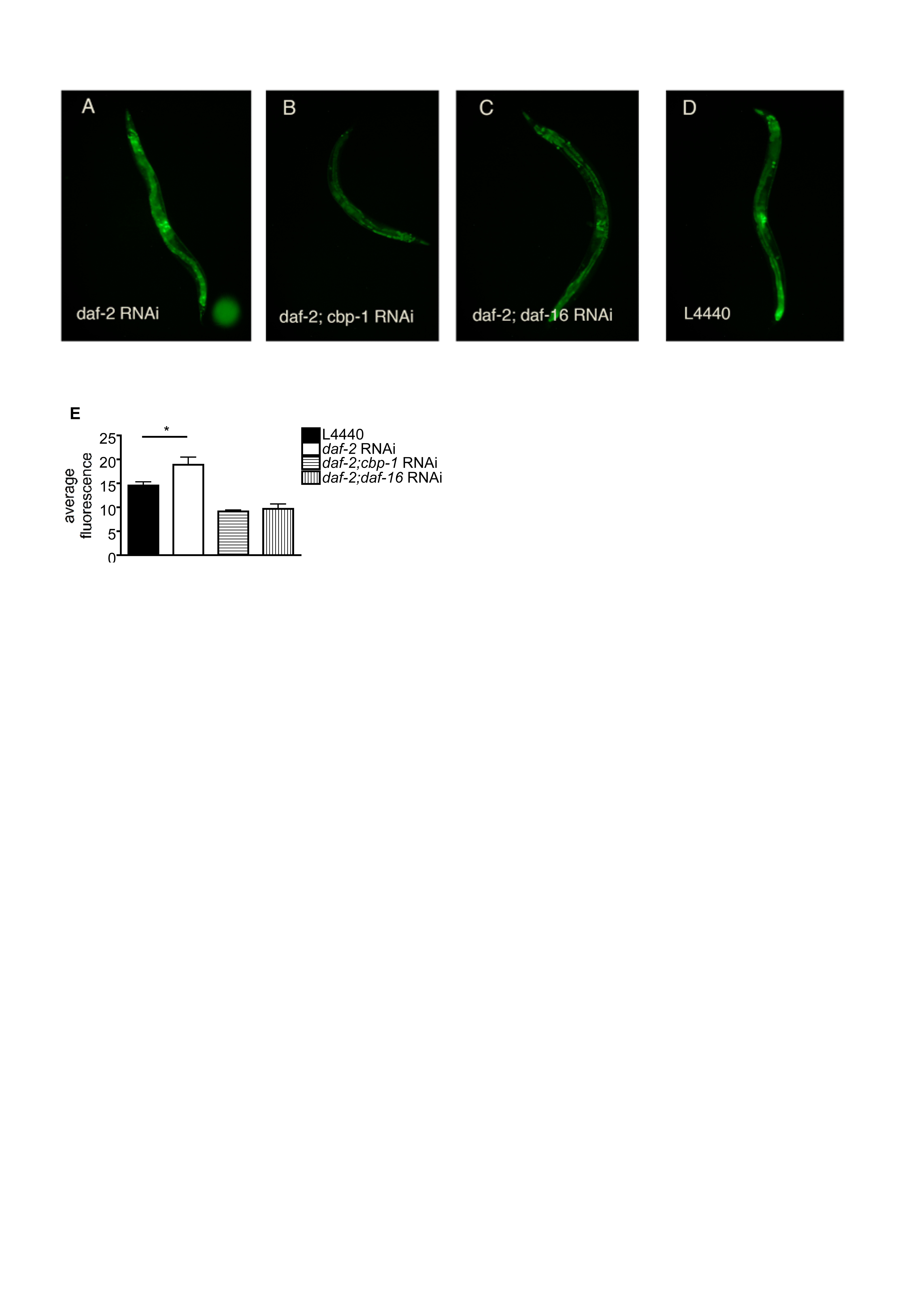

Supplement: Figure S5 — cbp-1 and daf-16 RNAi similarly block the induction of sod-3 by the daf-2 mutation. sod-3::GFP transgenic worms (CF1553) were fed with daf-2 dsRNA alone (A), daf-2 and cbp-1 dsRNA (B), daf-2 and daf-16 dsRNA (C), or control L4440 empty vector (D) at 20 C, and photographed by fluorescence microscope. RNAi dilution effect was controlled by 1∶1 dilution of daf-2 RNAi with L4440. (E) cbp-1 RNAi and daf-16 RNAi equally decreased sod-3 expression. Average GFP fluorescence was quantified using software imageJ and difference between groups was determined by student's t-test (*p<0.05, n = 5–7/group). (4.72 MB TIF) [file pbio.1000245.s005.tif]

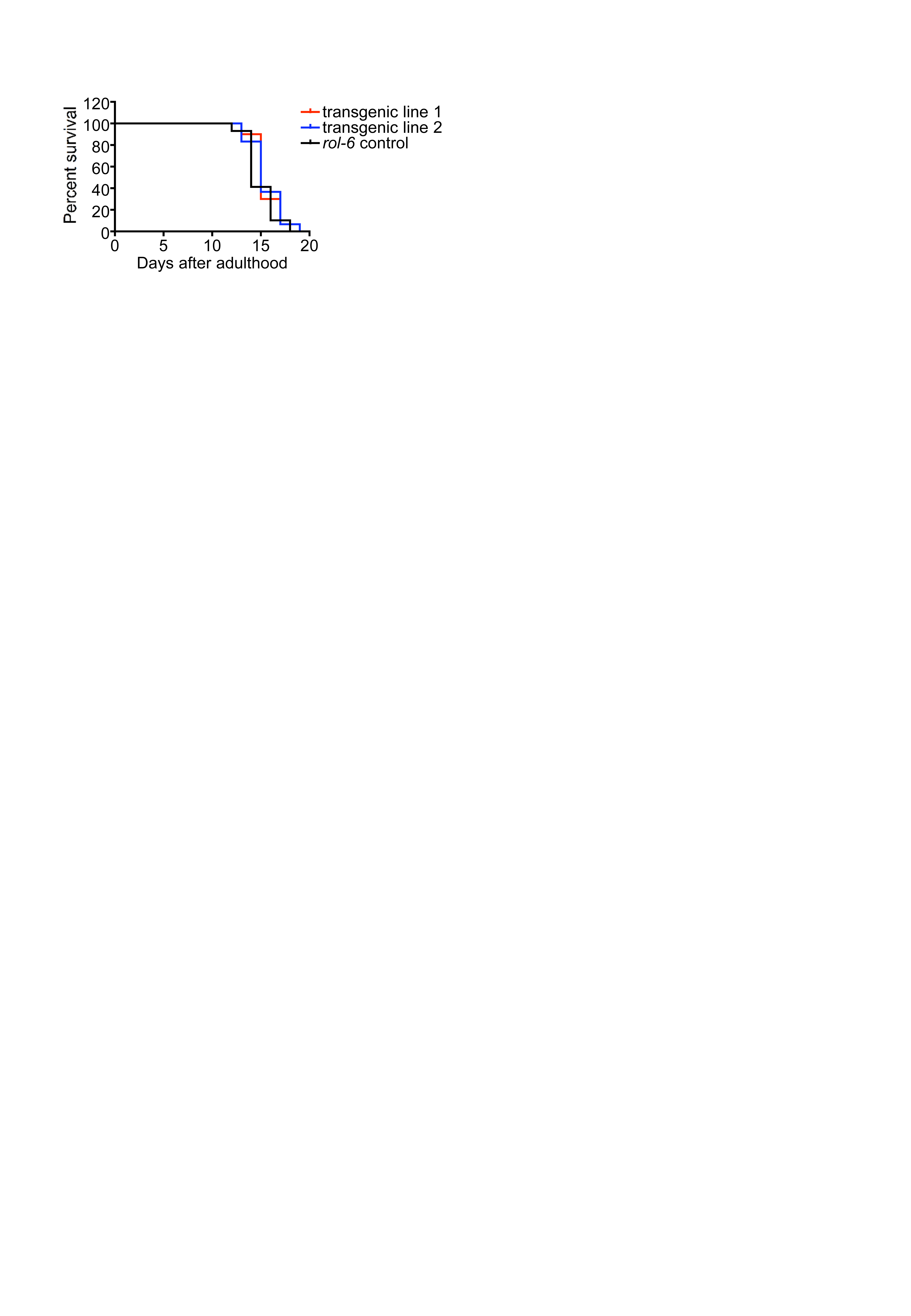

Supplement: Figure S6 — Overexpression of cbp-1 does not extend lifespan in N2 worms at 25°C. Transgenic worms were generated by co-microinjecting PCR product cbp-1::cbp-1 and rol-6 plasmid (pRF4) into N2 worms. Two independent transgenic lines were maintained and used in lifespan assay. Control worms were generated by injecting rol-6 plasmid alone. No lifespan extension was observed by cbp-1 overexpression (p = 0.11 line 1 versus control, p = 0.14 line 2 versus control). (2.65 MB TIF) [file pbio.1000245.s006.tif]

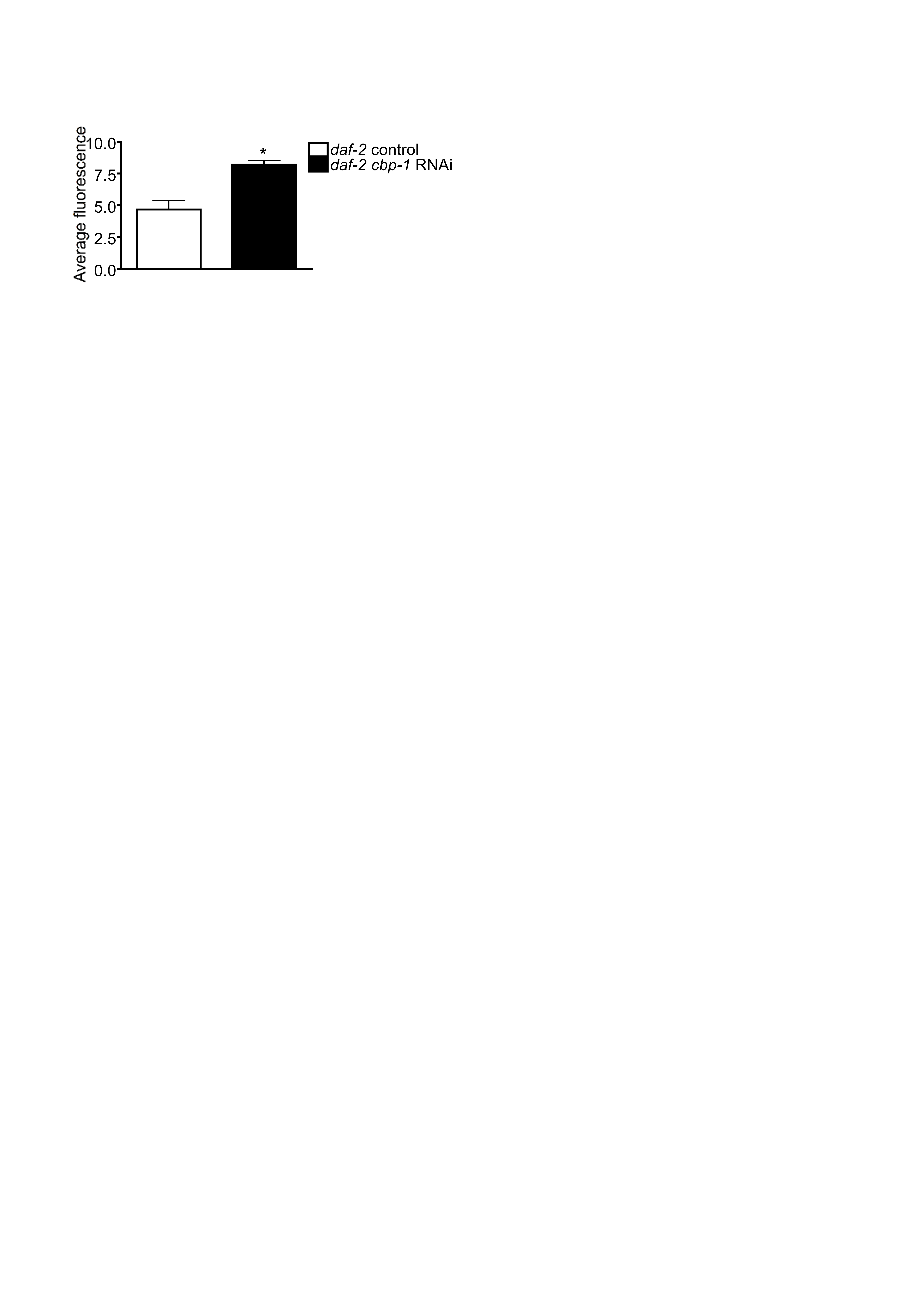

Supplement: Figure S7 — cbp-1 RNAi increases, rather than reverses, increased Nile Red staining produced by the daf-2 mutation. daf-2 mutant worms were fed with control bacteria or bacteria expressing cbp-1 dsRNA for 5 d at 25°C. Worms were stained with Nile Red dye and fluorescence was quantified software ImageJ. Data are presented as mean ± SEM (n = 5/group, p<0.05). (2.58 MB TIF) [file pbio.1000245.s007.tif]

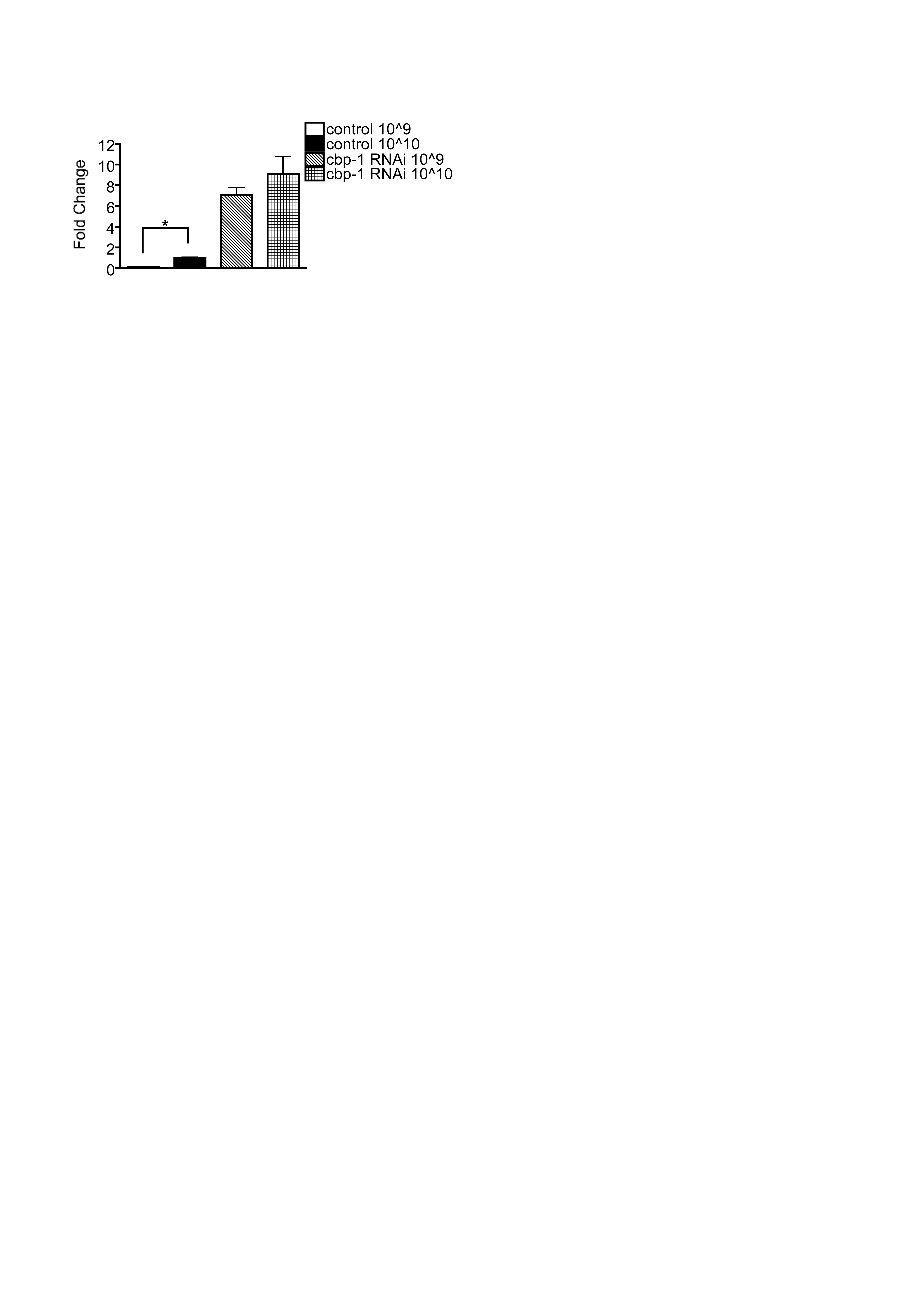

Supplement: Figure S8 — cbp-1 RNAi blocks the inhibition of F47G4.3, an ortholog of mammalian glycerophosphate dehydrogenase, by bDR. Data are presented as mean ± SEM (*p<0.05, n = 4–6/group). (2.81 MB TIF) [file pbio.1000245.s008.tif]

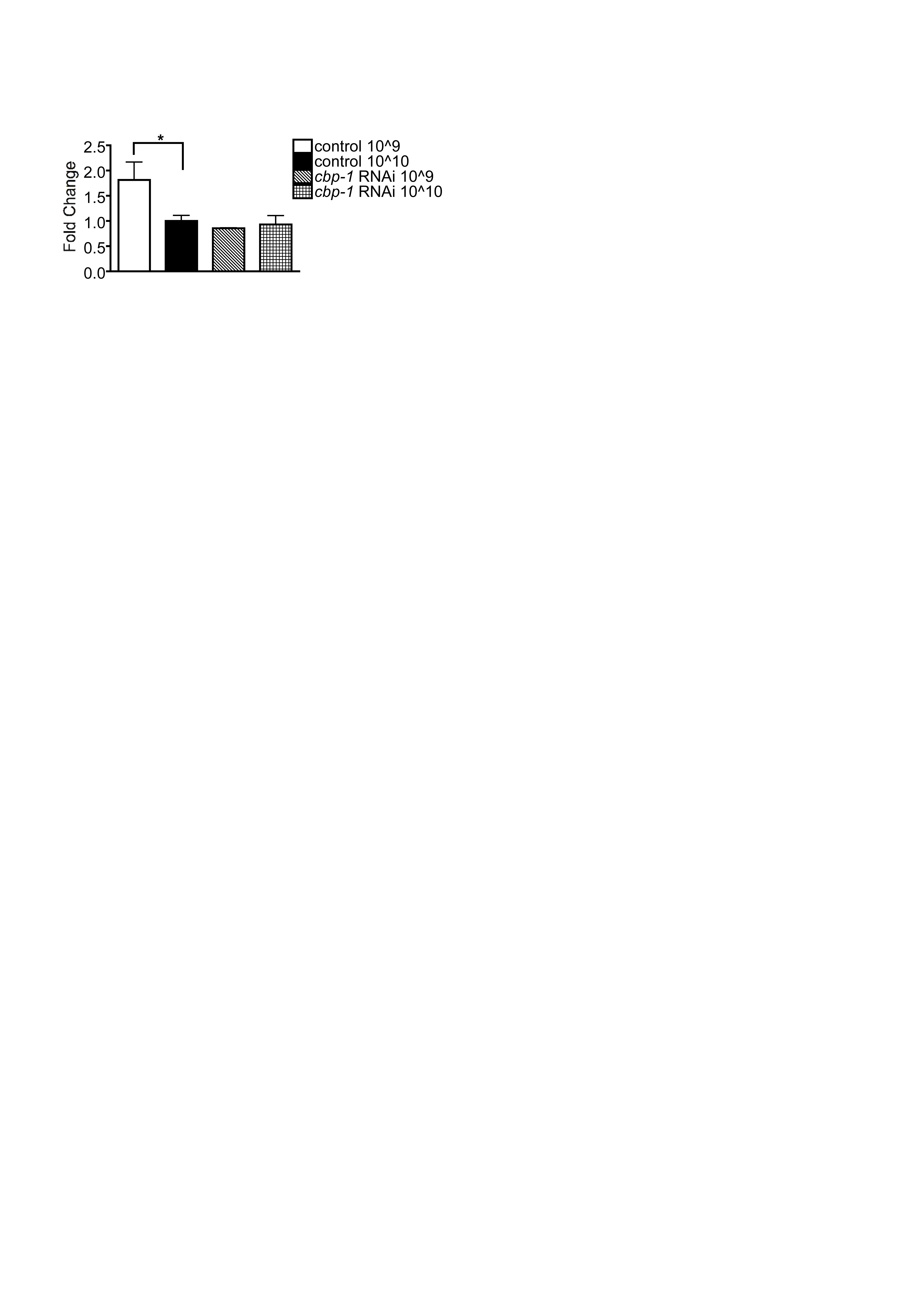

Supplement: Figure S9 — cbp-1 RNAi blocks the induction of ZK370.5, an ortholog of mammalian pyruvate dehydrogenase kinase, by bDR. Data are presented as mean ± SEM (*p<0.05, n = 4–6/group). (2.74 MB TIF) [file pbio.1000245.s009.tif]
