# Supplementary material for: Role of CBP and SATB-1 in Aging, Dietary Restriction, and Insulin-Like Signaling
Source: PLoS Biol. 2009 Nov 17;7(11):e1000245. doi: 10.1371/journal.pbio.1000245 (PMC2774267; doi:10.1371/journal.pbio.1000245)
Supplement: Table S1 — Summary of lifespan assays. (0.18 MB DOC) [file pbio.1000245.s010.doc]

**Supplementary table S1. Summary of lifespan assays**

| strains | Experiment | Average lifespan (± SD days) | # sample size/ # trials | p-value |
| --- | --- | --- | --- | --- |
| *rrf3* | L4440 10^8 20°C | 23.08±3.62 | 79/3 |  |
| *rrf3* | L4440 10^9 20°C | 33.59±6.48 | 244/4 |  |
| *rrf3* | L4440 10^10 20°C | 20.45±1.36 | 55/3 | P<0.0001 ^a^ |
| *rrf3* | *cbp-1* RNAi 10^8 20°C | 15.46±1.06 | 74/3 |  |
| *rrf3* | *cbp-1* RNAi 10^9 20°C | 15.62±2.11 | 244/4 |  |
| *rrf3* | *cbp-1* RNAi 10^10 20°C | 14.8±1.28 | 50/3 | P=0.0582 ^a^ |
| *rrf3* | *daf-16* RNAi 10^8 20°C | 17.54±3.01 | 53/2 |  |
| *rrf3* | *daf-16* RNAi 10^9 20°C | 19.90±0.51 | 74/2 |  |
| *rrf3* | *daf-16* RNAi 10^10 20°C | 13.30±1.75 | 35/2 | P<0.0001 ^a^ |
| *rrf3* | *dev-1* RNAi 10^8 20°C | 20.89±2.98 | 52/2 |  |
| *rrf3* | *dev-1* RNAi 10^9 20°C | 18.48±1.91 | 81/2 |  |
| *rrf3* | *dev-1* RNAi 10^10 20°C | 10.50±1.70 | 35/2 | P<0.0001 ^a^ |
| *rrf3* | *hsf-1* RNAi 10^8 20°C | 18.14±3.95 | 29/1 |  |
| *rrf3* | *hsf-1* RNAi 10^9 20°C | 16.47±2.42 | 43/1 |  |
| *rrf3* | *hsf-1* RNAi 10^10 20°C | 15.2±4.87 | 20/1 | P=0.9784 ^a^ |
|  |  |  |  |  |
| N2 | L4440 10^8 20°C | 21.83±6.34 | 29/1 |  |
| N2 | L4440 10^9 20°C | 34.61±6.96 | 46/1 |  |
| N2 | L4440 10^10 20°C | 20.9±2.79 | 20/1 | P<0.0001 ^a^ |
| N2 | *cbp-1* RNAi 10^8 20°C | 15.14±2.33 | 29/1 |  |
| N2 | *cbp-1* RNAi 10^9 20°C | 17.71±2.05 | 45/1 |  |
| N2 | *cbp-1* RNAi 10^10 20°C | 13.33±1.41 | 18/1 | P<0.0001 ^a^ |
| *daf-2 (e1370)* | L4440 10^8 20°C | 27.86±10.70 | 28/1 |  |
| *daf-2 (e1370)* | L4440 10^9 20°C | 48.36±12.70 | 45/1 |  |
| *daf-2 (e1370)* | L4440 10^10 20°C | 27.59±9.38 | 17/1 | P<0.0001 ^a^ |
| *daf-2 (e1370)* | *cbp-1* RNAi 10^8 20°C | 17.08±2.74 | 25/1 |  |
| *daf-2 (e1370)* | *cbp-1* RNAi 10^9 20°C | 19.31±4.80 | 45/1 |  |
| *daf-2 (e1370)* | *cbp-1* RNAi 10^10 20°C | 15.80±3.21 | 20/1 | P=0.0008 ^a^ |
|  |  |  |  |  |
| *rrf3* | L4440 agar 20°C | 21.70±3.69 | 40/1 |  |
| *rrf3* | *cbp-1* RNAi agar 20°C | 13.50±1.85 | 40/1 | P<0.0001 ^b^ |
| *eat-2 (ad1113)* | L4440 agar 20°C | 25.90±4.51 | 40/1 |  |
| *eat-2 (ad1113)* | *cbp-1* RNAi agar 20°C | 13.63±1.55 | 38/1 | P<0.0001 ^b^ |
|  |  |  |  |  |
| N2 | L4440 agar 20°C | 18.88±2.95 | 149/3 |  |
| N2 | *cbp-1* RNAi agar 20°C | 15.50±2.35 | 48/1 | P<0.0001 ^b^ |
| *glp-1 (e2141)* | L4440 agar 20°C | 23.29±2.65 | 49/1 |  |
| *glp-1 (e2141)* | *cbp-1* RNAi agar 20°C | 17.94±2.31 | 51/1 | P<0.0001 ^b^ |
| *clk-1 (e2519)* | L4440 agar 20°C | 28.47±5.47 | 38/1 |  |
| *clk-1 (e2519)* | *cbp-1* RNAi agar 20°C | 15.09±1.58 | 44/1 | P<0.0001 ^b^ |
| *mev-1 (kn1)* | L4440 agar 20°C | 18.09±4.04 | 46/1 |  |
| *mev-1 (kn1)* | *cbp-1* RNAi agar 20°C | 14.00±2.27 | 46/1 | P<0.0001 ^b^ |
|  |  |  |  |  |
| *daf-2 (e1370)* | L4440 agar 25°C | 36.26±6.47 | 187/4 |  |
| *daf-2 (e1370)* | *cbp-1* RNAi agar 25°C | 16.90±3.95 | 174/4 | P<0.0001 ^b^ |
| *daf-2 (e1370)* | *daf-16* RNAi agar 25°C | 14.20±2.29 | 177/4 |  |
| *daf-2 (e1370)* | *dev-1* RNAi agar 25°C | 27.59±6.81 | 49/1 |  |
| *daf-2 (e1370)* | *hsf-1* RNAi agar 25°C | 20.31±4.99 | 49/1 |  |
| N2 | L4440 agar 25°C | 13.92±1.49 | 89/2 |  |
| N2 | *cbp-1* RNAi agar 25°C | 9.06±1.01 | 88/2 | P<0.0001 ^b^ |
| N2 | *daf-16* RNAi agar 25°C | 10.67±0.95 | 86/2 |  |
|  |  |  |  |  |
| *rrf3* | *cbp-1* RNAi agar 25°C | 11.63±1.00 | 102/1 |  |
| *rrf3* | *daf-16* RNAi agar 25°C | 11.36±1.36 | 100/1 |  |
| *rrf3* | *dev-1* RNAi agar 25°C | 11.38±1.16 | 100/1 |  |
| *rrf3* | *hsf-1* RNAi agar 25°C | 10.05±1.15 | 40/1 |  |
| *rrf3* | L4440 agar 25°C | 15.38±2.13 | 127/3 |  |
| *rrf3* | L4440 to axenic media 25°C | 23.14±3.34 | 128/3 | P<0.0001 ^b^ |
| *rrf3* | *cbp-1* RNAi to L4440 25°C | 11.10±1.36 | 127/3 |  |
| *rrf3* | *cbp-1* RNAi to axenic media 25°C | 12.60±1.58 | 127/3 | P<0.0001 ^b^ |
|  |  |  |  |  |
| *rrf3* | L4440 agar 16°C | 22.70±4.38 | 40/1 |  |
| *rrf3* | *cbp-1* RNAi agar 16°C | 16.05±2.46 | 40/1 | P<0.0001 ^b^ |
|  |  |  |  |  |
| *daf-16 (m26)* | L4440 agar 20°C | 12.84±2.11 | 50/1 |  |
| *daf-16 (m26)* | *cbp-1* RNAi agar 20°C | 12.20±1.37 | 51/1 | P=0.0714 ^b^ |
|  |  |  |  |  |
| *rrf3* | *cbp-1*, *daf-16* RNAi (1:1) agar 20°C | 14.75±1.47 | 48/1 |  |
| *rrf3* | *cbp-1* RNAi, L4440 (1:1) agar 20°C | 15.59±1.63 | 49/1 | P=0.009 |
| *rrf3* | *daf-16* RNAi, L4440 (1:1) agar 20°C | 14.65±2.25 | 49/1 | P=0.803 |
|  |  |  |  |  |
| N2 | Agar 20°C | 20.84±4.71 | 29/1 |  |
| N2 | Agar 20°C 5mM NaB | 25.20±4.15 | 31/1 | P<0.0001 |
| N2 | Agar 20°C 150ng/ul TSA | 23.21±4.01 | 29/1 | P=0.0169 |
| N2 | L4440 agar 20°C | 19.12±2.44 | 50/1 |  |
| N2 | L4440 agar 20°C 5mM NaB | 21.08±2.91 | 50/1 | P=0.0006 |
| N2 | *cbp-1* RNAi agar 20°C | 14.16±1.46 | 50/1 |  |
| N2 | *cbp-1* RNAi agar 20°C 5mM NaB | 14.56±1.47 | 50/1 | P=0.1770 |
| N2 | L4440 10^9 20°C | 26.87±6.11 | 45/1 |  |
| N2 | L4440 10^9 20°C 5mM NaB | 35.60±6.70 | 43/1 | P<0.0001 |
| N2 | *cbp-1* RNAi 10^9 20°C | 16.36±2.08 | 45/1 |  |
| N2 | *cbp-1* RNAi 10^9 20°C 5mM NaB | 16.48±2.28 | 45/1 | P=0.6816 |
|  |  |  |  |  |
| N2 | L4440 10^9 20°C | 33.40±8.36 | 47/1 |  |
| N2 | L4440 10^9 20°C 2mM NaB | 30.23±9.28 | 43/1 | P=0.0874 |
| N2 | *cbp-1* RNAi 10^9 20°C | 16.52±1.93 | 42/1 |  |
| N2 | *cbp-1* RNAi 10^9 20°C 2mM NaB | 17.56±2.17 | 45/1 | P=0.0242 |

1. Vs. 10^9 cells/ml
2. Vs. agar control
