# Supplementary material for: Role of CBP and SATB-1 in Aging, Dietary Restriction, and Insulin-Like Signaling
Source: PLoS Biol. 2009 Nov 17;7(11):e1000245. doi: 10.1371/journal.pbio.1000245 (PMC2774267; doi:10.1371/journal.pbio.1000245)
Supplement: Table S2 — Gompertz values for lifespan assays. (0.04 MB DOC) [file pbio.1000245.s011.doc]

Supplementary table S2. Gompertz values for lifespan assays

|  | G | A | R^2^ | Sample size (n) |
| --- | --- | --- | --- | --- |
| *rrf3* Ad-lib agar 20°C | 0.4343 | 0.0002965 | 0.9892 | 99 |
| *rrf3* L4440 10^9 20°C | 0.1949^a^ | 0.0001881 | 0.9936 | 120 |
| *rrf3* *cbp-1* RNAi 10^9 20°C | 0.7788^a,b^ | 8.673e-006 | 0.9914 | 121 |
| *rrf3* L4440 agar 25°C | 0.6133 | 5.075e-005 | 0.9989 | 107 |
| *daf-2* L4440 agar 25°C | 0.2454^c^ | 3.613e-005 | 0.9944 | 90 |
| *daf-2 cbp-1* RNAi agar 25°C | 0.3460^c,d^ | 0.0004293 | 0.9915 | 99 |
| *daf-2 daf-16* RNAi agar 25°C | 0.3051^c,d^ | 0.001102 | 0.9910 | 88 |

1. significantly different from *rrf3* ad-lib agar 20C (p<0.01)
2. significantly different from *rrf3* L4440 10^9 20C (p<0.01)
3. significantly different from *rrf3* L4440 agar 25C (p<0.01)
4. significantly different from *daf-2* L4440 agar 25C (p<0.01)
